# The role of ABC transporters in the human lung epithelium—insights from and limitations of current in vitro cell models

**DOI:** 10.1007/s44164-025-00091-w

**Published:** 2025-08-14

**Authors:** Sina Simon, Carina Cantrill, Claus-Michael Lehr

**Affiliations:** 1 Roche Pharma Research and Early Development, Pharmaceutical Sciences, Roche Innovation Center Basel, F. Hoffmann-La Roche Ltd, Grenzacherstrasse 124, 4070 Basel, Switzerland; 2 Department of Pharmacy and Pharmaceutical Science Hub (PSH), Saarland University, Campus E8.1, 66123 Saarbrücken, Germany; 3 Helmholtz Centre for Infection Research, Helmholtz Institute for Pharmaceutical Research Saarland, Campus E8.1, 66123 Saarbrücken, Germany

**Keywords:** Pulmonary drug distribution, Membrane transporter, Active efflux, Bidirectional permeability study

## Abstract

ATP-binding cassette (ABC) drug transporter proteins are expressed at the level of the human pulmonary epithelium. This protein superfamily is known to clinically affect the pharmacokinetics of their substrates in tissues such as the intestine or liver. In contrast, it is not yet entirely understood to what degree ABC transporters contribute as drivers of pulmonary distribution. In recent decades, a number of in vitro studies have been conducted to elucidate the role of ABC transporter in the human lung including human derived cell lines, primary cells and human lung tissue. Results indicate the functional expression of ABC transporters in vitro. However, ABC expression patterns vary across the different cells mimicking upper and lower airways. Since the lung is a target for a variety of drug classes, it is of importance to understand the influence of ABC transporters to pulmonary drug disposition and local drug exposure levels. This review gives an overview over conducted in vitro studies that do not focus only on ABC transporters’ expression but also on assessing their functional activity. Moreover, we pinpoint important aspects that need to be taken into account when conducting these in vitro bidirectional transporter efflux studies. This contributes to a better understanding of the importance of these pulmonary in vitro studies and provides guidance on how to interpret and conduct these studies.

## Introduction

There are three groups of drug transporter families, which appear to play a role in the drug disposition of xenobiotics in the human body: ATP-binding cassette (ABC), solute carrier (SLC) and solute carrier organic anion (SLCO) transporters [1]. In the lung, mainly ABC and SLC transporters are found [2]. These transporter proteins were described to be expressed in the membrane of the lung epithelium in both upper and lower airways, on either the apical lung lumen facing side or the basolateral side facing the pulmonary capillaries. Few studies have also reported their presence in the pulmonary endothelial and local immune cells [3].

Within the scope of this review, the focus will lay on ABC transporters. ABC transporters are known to be expressed in a variety of human tissues where they have a significant clinical impact on the absorption, distribution and elimination of their substrates. This can result in limited intestinal absorption, enhanced biliary/renal excretion and/or reduced tissue distribution of their substrates. Therefore, numerous drug-drug interactions are reported for their substrates [4, 5]. However, the clinical relevance of ABC transporters in the human lung is not entirely understood [2].

ABC transporters are proteins that are capable of extruding a large and diverse range of structurally unrelated compounds, in an energy-dependent manner against a concentration gradient across a cellular membrane. This energy is provided by ATP hydrolysis and thus classifies these transmembrane proteins as primary active transporters. Prominent members of this family are P-glycoprotein (P-gp, multidrug resistance protein 1 (MDR1)), multidrug resistance associated proteins (MRPs) and breast cancer resistance protein (BCRP) [6].

A detailed summary on the expression of drug transporters in the lung mucosa, including MDR1, MRP1 and BCRP, is described in reviews by Nickel et al. and Gumbleton et al. [2, 3]. They showed that findings from different studies are not always consistent, meaning some studies failed to detect a specific transporter protein in a certain cell type whereas others were able to detect it.

This review will provide a summary of in vitro studies, using human lung epithelial cells, which include a functionality assessment of the expressed ABC transporters (MDR1, MRP1 and BCRP) and refers to challenges of conducting and interpreting such studies.

## An overview of the lung biology and in vitro mimicking cells

### Anatomical structure of the human airways

**Fig. 1:**
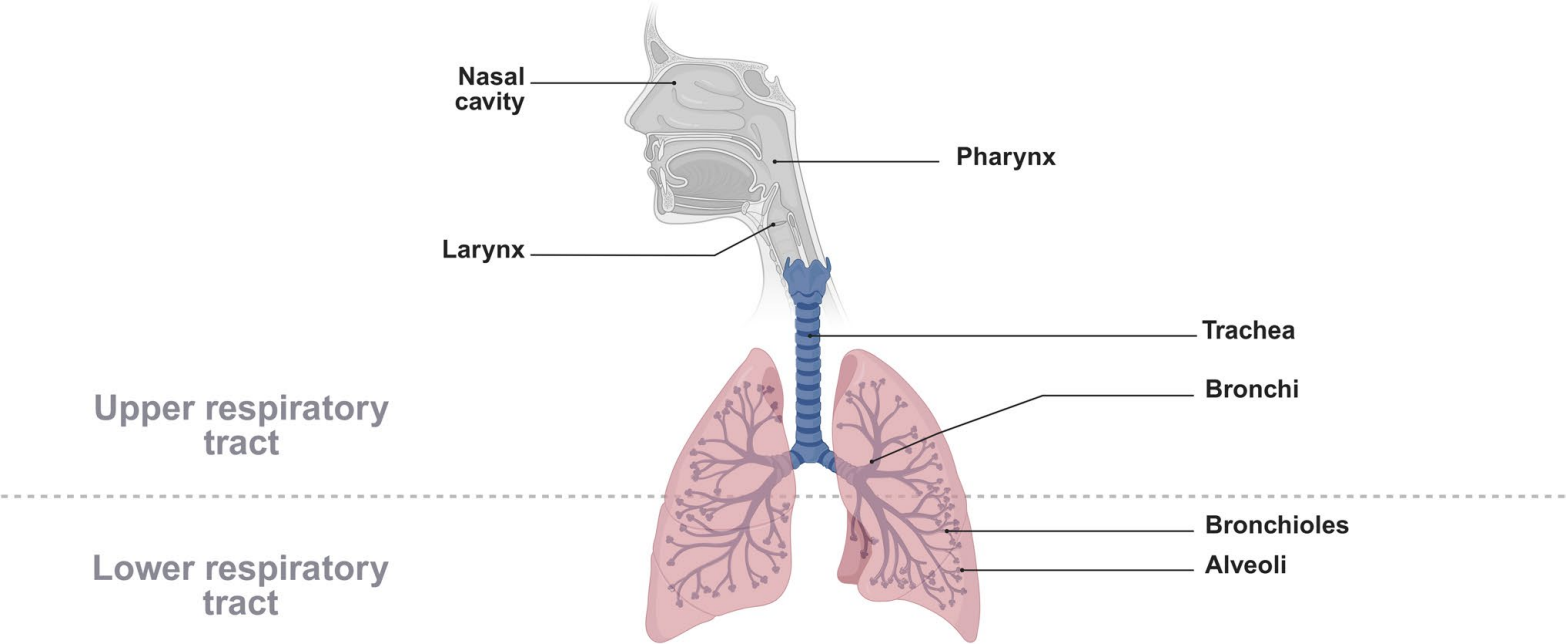
The structure of the human respiratory tract divided into upper and lower parts. By breathing in, air enters the human body mainly via the nasal cavity, passing pharynx, larynx and the trachea. Due to bifurcation of the trachea, air can flow into the right and left lung and reach bronchial, bronchiolar and final alveolar space where the alveolar sacs are located for gas exchange [7, 8]. Image created in https://BioRender.com

The human respiratory tract is divided into the upper and lower airways. The upper or conducting airways consist of the nasal cavity, pharynx, larynx, trachea, bronchi and bronchiole. Respiratory bronchioles and alveoli constitute the lower respiratory system [7]. A graphical representation of the human respiratory tract is shown in Fig. 1.

Starting at the trachea, the airway bifurcates up to 23 times down to the alveoli, which causes a descending reduction in airway diameter from 3–5 mm in the bronchial region down to 250 µM in the alveolar region. The bifurcation causes, at the same time, an almost 50-fold increase in surface area from 2 to 3 m^2^ in conducting airways to around 100 m^2^ in the alveolar region [8, 9].

The whole airways are covered by a thin fluid layer that is called epithelial lining fluid (ELF) [10]. Along the airways from proximal to distal region, the cellular morphology and structure are highly variable and adapted to the functional need of the respective region. The trachea-bronchial region is characterised by a pseudostratified columnar epithelium consisting of different cell types including beating ciliated cells, mucus-producing and secreting goblet cells, neuroendocrine cells and basal cells. The bronchiolar section has a similar composition as the trachea-bronchi, consisting of ciliated, goblet, club cells (formerly known as clara cells), neuroendocrine and basal cells. In contrast, the epithelium is simple columnar, changing to a simple cuboidal one in the respiratory bronchioles. The alveolar epithelium is classified as simple squamous epithelium and is covered by the alveolar epithelial cells type I and II (referred to as alveolar pneumocytes type I and II). The thin type I pneumocytes cover more than 95% of the alveolar surface and are in charge of the gas exchange. Type II pneumocytes secrete surfactant, which covers the alveolar surface and reduces the surface tension and further stabilises the alveoli during the breathing process. They also serve as progenitor cells for type I pneumocytes. Alveolar macrophages are the local immunocompetent cells and engulf particles [11–15].

### Human pulmonary epithelial cellsin vitro

Over the recent decades, many in vitro studies to elucidate the role of drug transporters in the pulmonary epithelium have been conducted. Transporter expression was investigated in human pulmonary epithelium-derived cell lines and primary cells from all regions of the lung [2]. Detailed reviews on in vitro systems, including co-cultures that can be used to investigate pulmonary disposition, have been published [16, 17].

**Fig. 2:**
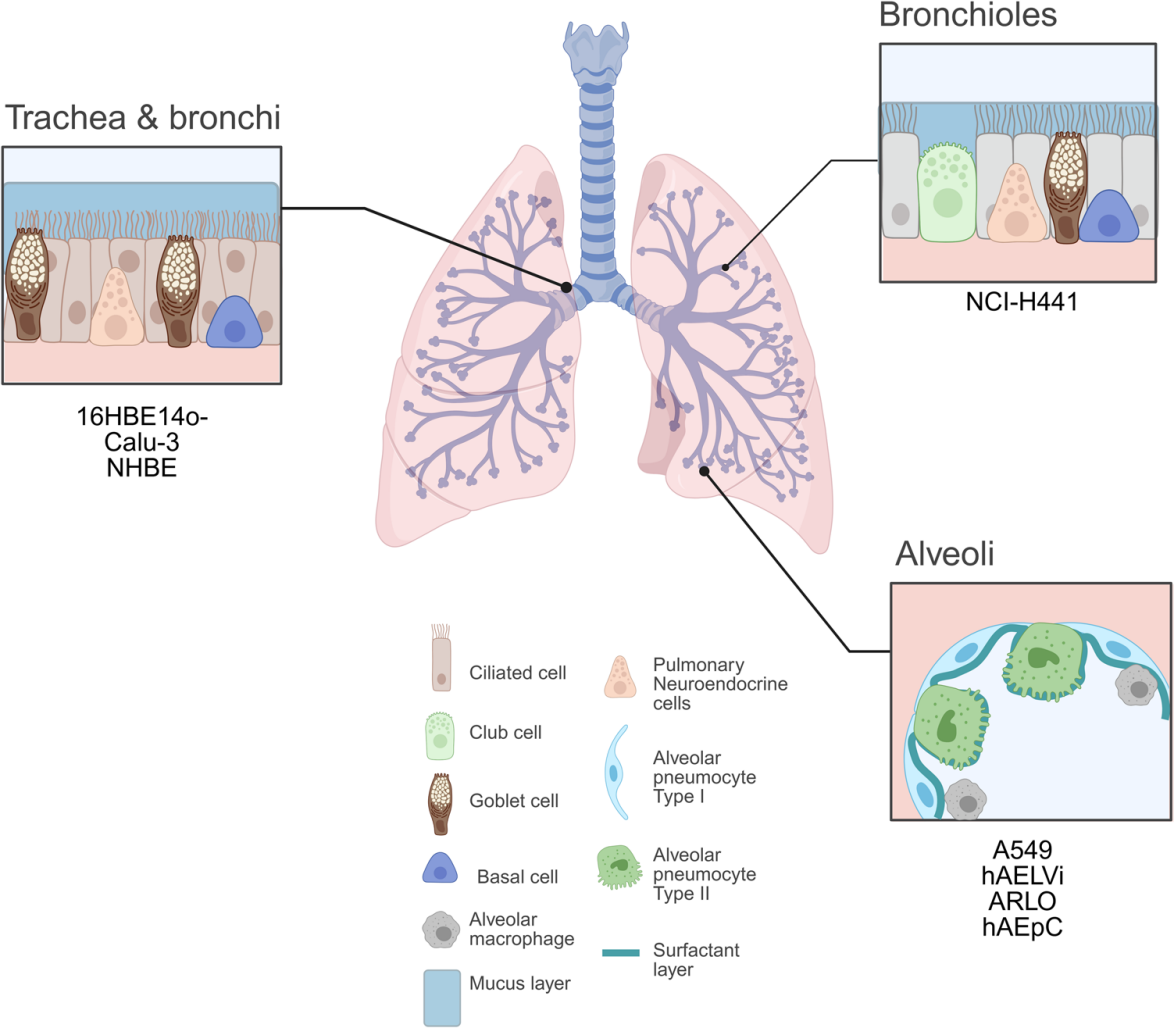
The cellular composition of the human airways in the different regions and derived cells that are mimicking the respective region. Image created in https://BioRender.com

The various in vitro cell models, mimicking the human lung epithelium, to which this review will refer to in terms of ABC transporters’ functional activity studies are shown in Fig. 2.

Lung epithelial cells are usually cultured on porous membrane inserts to perform in vitro pulmonary distribution studies. Cells can be cultured under submerged conditions (liquid–liquid interface (LLI), medium on both sides of the insert) or at the air–liquid interface (ALI, medium only at the basolateral side established after cells reach confluence) [18].

An important prerequisite to conduct these pulmonary distribution studies is that the cells form a tight barrier [16]. Therefore, transepithelial electrical resistance (TEER) is often measured to verify barrier integrity [19].

Table 1 provides an overview on conducted in vitro studies that aimed to mimic the different regions of the human lung epithelium, using a variety of human lung-derived cell lines and primary cells. The cells, that are in scope of this review and as shown in Fig. 2, will be described in more details regarding their characteristics and ability to form a tight barrier when cultured in vitro. As mentioned above, the formation of a tight barrier is key when assessing the permeability of a compound across a biological barrier and by this characterising active transporter processes. Therefore, reported TEER values for the individual cell lines and primary cells are shown in Table 1.

**Table 1:** Overview of origin and reported transepithelial electrical resistance (TEER) of cell lines and primary cells derived from the different regions of the human lung epithelium. The TEER values are reported for air–liquid interface (ALI) and liquid–liquid-interface (LLI) cultured cells

Mimicked region	Cell name	Cell type	Origine	TEER [Ω*cm^2^]	Characteristics on barrier formation	References
Trachea-bronchial	16HBE14o-	Immortalised cell line	Immortalisation by SV40 T antigen transformation from primary bronchial epithelial cells	~ 130–250 (ALI) ~ 800 (LLI)	If cultured at ALI, more than 10-cells-thick layer was formed, whereas partial monolayer to multilayer (up to 5 layers) formed at LLI	[20–22]
	Calu-3	Cancer cell line	Isolated from a lung adenocarcinoma patient of bronchial submucosal gland origin	~ 400–800 (ALI) > 1000 (LLI)	Culture conditions (ALI vs. LLI) appear to affect cells morphology and barrier formation properties	[23–26]
	Normal bronchial epithelial cells (NHBE)	Primary cells	Isolated from human bronchial lung tissue	~ 800–900 (ALI) ~ 1600 (LLI)	If cultured at ALI, cells differentiate into a pseudostratified epithelium with mucus production and cilia beating, however formation of multilayers at ALI reported	[27–29]
Bronchiolar	NCI-H441	Cancer cell line	Isolated from pericardial fluid of a patient with papillary lung adenocarcinoma with features of club cells and alveolar pneumocytes type II	300–450 (ALI) ~ 1000 (LLI)	Culture medium needs to be supplemented with dexamethasone, otherwise TEER < 100 Ω*cm^2^ were reported	[30–32]
Alveolar	A549	Cancer cell line	Alveolar adenocarcinoma with hallmarks of type II alveolar pneumocytes (lamellar bodies)	< 60 (ALI & LLI)	Lack of proper barrier formation, not well suited for performing drug permeation studies in vitro	[30, 33–35]
	hAELVi	Immortalised cell line	Immortalisation via lentivirus transfection from primary alveolar epithelial cells	~ 1500–3000 (ALI) ~ 500–2000 (LLI)	Tight barrier formation observed at both ALI and LLI after 14 days of culture	[36, 37]
	Arlo	Immortalised cell line	Derived from hAELVi via single-cell printing	~ 3000 (ALI) ~ 2000 (LLI)	Tight barrier formation observed at both ALI and LLI after 14 days of culture	[38]
	Type I-like alveolar pneumocytes (hAEpC)	Primary cells	Isolated from human alveolar tissue	> 2000 (ALI) ~ 1000 (LLI)	Peak of TEER reached after ~ 6 to 8 days, followed by decline in TEER	[36, 38, 39]

As we will report in the following, all of these cells have already been characterised in vitro to some extent for their expression and functional activity of ABC transporter proteins. However, the reported expression levels differ across the investigated models and there remains the open question if one of these models is an appropriate tool to capture ABC transporter-mediated processes at the human pulmonary epithelium.

## Assessing the functional activity of ABC transporters within cells mimicking the human lung epithelium

### Setup of bidirectional transport studies

**Fig. 3:**
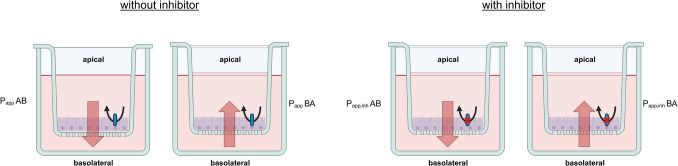
Schematic representation of the bidirectional approach to assess the functional activity of an active efflux transporter. The oval represents the expression of a hypothetical efflux transporter at the apical membrane. Cells are seeded onto the porous inserts and over time form a tight and polarised monolayer. The compound of interest is applied to the apical side and the amount reaching the basolateral reservoir is quantified by calculating the *P*_app_. This is done in absence and presence of transporter-specific inhibitor (*P*_app_ AB; *P*_app,inh_ AB). The same is performed in basolateral to apical direction where the compound is dosed to the basolateral reservoir and the amount of drug reaching the apical insert is measured, in absence and presence of inhibitor (*P*_app_ BA and *P*_app,inh_ BA). Image created in https://BioRender.com

The expression of ABC transporters (including MDR1, MRP1 and BCRP) in the in vitro lung cell models has been characterised utilising an array of methods such as polymerase chain reaction (PCR), microarrays, Western Blotting, mass spectrometry-based proteomic approaches and immunofluorescent staining [40–43]. However, to affect pulmonary drug disposition, drug transporters need to be functionally expressed at the cell membrane surface. To investigate transporter functionality, bidirectional transport studies with known substrates and inhibitors of the respective drug transporter are performed. Figure 3 shows the experimental setting for these bidirectional transporter studies using cells grown on porous membrane inserts.

The apparent permeability coefficient (*P*_app_) describes the amount of compound that crosses the membrane in a defined period of time. Absorptive (apical to basolateral, *P*_app_ AB) and secretory permeability (basolateral to apical,* P*_app_ BA) across the cellular layer are then compared to assess if there is asymmetry in absence of inhibitor, which is an indicator for active mediated transport [42, 44].

1 $${P}_{app}= \frac{dQ}{dt}* \frac{1}{A* {C}_{0}}$$

Equation 1 calculates the apparent permeability (*P*_app_), where dQ/dt represents the amount of test compound transported over time, *A* represents the filter surface area and *C*_ο_ is the initial compound concentration [45].

For ABC transporters active at the apical membrane, an efflux ratio (ER; apparent permeability from basolateral to apical site is divided by apparent permeability from apical to basolateral side) can be derived. An ER ≥ 2 is set as a threshold to classify a transporter substrate. If the asymmetry in transport is abolished by at least 50% in the presence of a transporter-specific inhibitor (by comparing Papp,inh AB and Papp,inh BA), this is considered confirmative of the functional involvement of the transporter protein. These guidance rules are recommended by Regulatory authorities (FDA and EMA) [5].

2 $$ER= \frac{{P}_{app} BA}{{P}_{app} AB}$$

Equation 2 calculates the efflux ratio (ER). The secretory permeability (*P*_app_ BA) is divided by the absorptive permeability (*P*_app_ AB) [45].

An inverse of the ER, the so-called uptake ratio (UR), was introduced in a study where the basolateral activity of an ABC transporter (MRP1) was assessed [42]. This ratio is calculated by dividing the apparent permeability from apical to basolateral side by the apparent permeability from basolateral to apical side. If the *P*_app_ AB is significantly higher than *P*_app_ BA, this hints to a basolateral expression of an active ABC transporter.

3 $$UR= \frac{{P}_{app} AB}{{P}_{app} BA}$$

Equation 3 calculates the uptake ratio (UR). The absorptive permeability (*P*_app_ AB) is divided by the secretory permeability (*P*_app_ BA) [42].

As previously mentioned, cells seeded onto insert plates need to form a tight and polarised layer to perform in vitro transcellular studies. This is often verified by measuring TEER values directly before and after conducting these kinds of experiments [46]. Membrane integrity can also be assessed by measuring the apparent permeability of small paracellular makers such as mannitol/dextran or fluorescent markers (e.g. Lucifer yellow/fluorescein sodium). For these extracellular markers, a low permeability coefficient is expected if the cell barrier is tight (e.g. a cut-off of 25 nm/s was reported for Lucifer yellow) [45, 47].

**Fig. 4:**
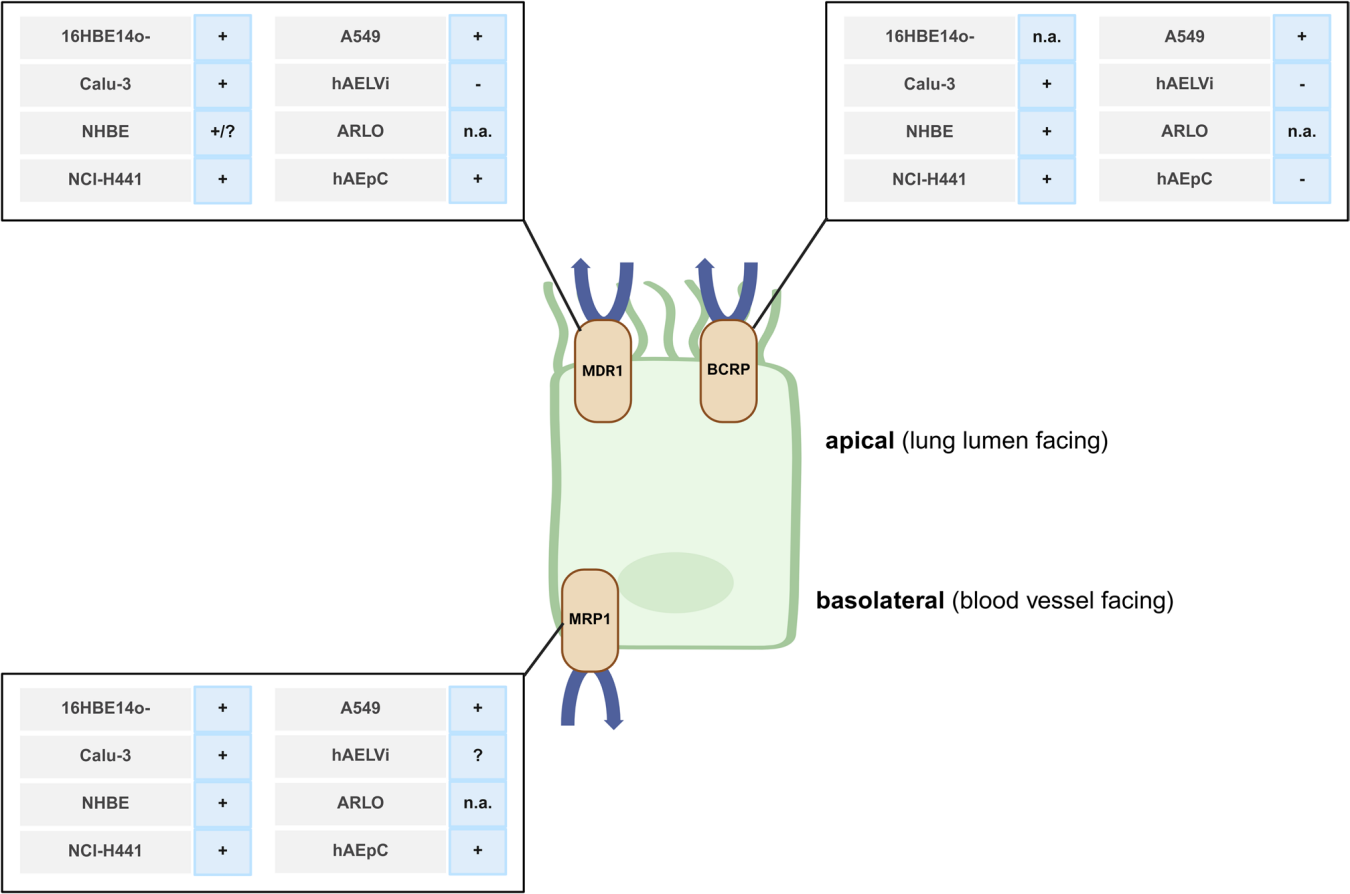
Schematic representation of the proposed location of ABC transporter expression in the human lung epithelium and the absence/presence of ABC transporters’ active efflux in the respective cell lines and primary cells mimicking the human lung epithelium. Symbols behind the cells’ name indicate the presence (+) or absence (−) of active efflux of either MDR1, MRP1 and BCRP, inconclusive data (?) or the non-availability of data (n.a.) and are based on the data summarised in Tables 1, 2, and 3*.* Image created in https://BioRender.com

The following section will provide an overview of the conducted in vitro studies profiling the functional activity of MDR1, MRP1 and BCRP in human lung epithelial cells. Figure 4 illustrates a summary of these studies and indicates the presence or absence of active efflux of the three ABC transporters in the different lung cells.

**Table 2:** Summary and outcome of in vitro studies conducted to investigate MDR1 efflux in cells mimicking the human lung epithelium. For each study, additional details on culture conditions and used transporter substrates and inhibitors are provided. N/A stands for non-available

Cell type	Study type	Culture condition	Culture time	Culture format	Probe substrate	Inhibitor	Outcome	Reference
16HBE14o-	Bidirectional transport	LLI	7 d	Inserts	Rh123	Verapamil	ER ~ 3, in presence of inhibitor reduced to an ER ~ 1	[52]
Calu-3	Bidirectional transport	ALI	21 d^a^	Inserts	Rh123	PSC833	ER ~ 7, in presence of inhibitor reduced to an ER ~ 2.8	[42]
Calu-3^b^	Bidirectional transport	ALI	21 d	Inserts	[^3^H]-digoxin	PSC833	ER ~ 11.4^c^, in presence of inhibitor reduced to an ER ~ 1.2^c^	[49]
Calu-3	Bidirectional transport	ALI	13–15 d	Inserts	Rh123	Cyclosporine A	ER ~ 10, in presence of inhibitor reduced to an ER < 1	[56]
Calu-3	Bidirectional transport	ALI	21 d	Inserts	Rh123	Verapamil	ER ~ 2.4, in presence of inhibitor reduced to an ER ~ 1	[25]
NHBE	Bidirectional transport	ALI	21 d	Inserts	[^3^H]-digoxin	Not tested	ER ~ 2.3 but repetition in another batch of same donor led to ER ~ 1^d^	[49]
NHBE	Bidirectional transport	ALI	6–8 d	Inserts	Rh123	Verapamil	ER ~ 3.0, in presence of inhibitor reduced to an ER ~ 1.7	[27]
EpiAirway™	Bidirectional transport	ALI	21 d	Inserts	Rh123	PSC833	ER ~ 1.3 in absence and presence of inhibitor	[42]
NCI-H441	Bidirectional transport	LLI	8 d	Inserts	Rh123	LY335979	ER ~ 3.6, in presence of inhibitor reduced to an ER < 1	[31]
A549	Cellular accumulation study	Liquid-covered	5 d	Well plate	Rh123	Cyclosporine A, verapamil	~ 50% reduction in intracellular concentration of Rh123 within 30 min after loading; not observed in presence of inhibitors	[60]
hAELVi	Bidirectional transport	ALI	21 d	Inserts	Rh123	PSC833	ER ~ 1.0 in absence and presence of inhibitor	[61]
EpiAlveolar™	Bidirectional transport	ALI	N/A^e^	Inserts	Rh123	PSC833	ER ~ 1.3 in absence and presence of inhibitor	[61]
hAEpC	Bidirectional transport	ALI	8 d	Inserts	Rh123	Verapamil	ER ~ 3.1, in presence of inhibitor reduced to an ER ~ 1.0	[63]

^a^Same experiment was performed when cells were cultured for 7 days on inserts [42]

^b^Calu-3 cells tested at both low (#25–30) and high (#45–50) passage numbers [49]

^c^ER values shown for Calu-3 cells at low passage [49]

^d^Potential issue in terms of cell monolayer integrity might have caused the disconnect [49]

^e^Exact culture time not defined [61]

**Table 3:** Summary and outcome of in vitro studies conducted to investigate MRP1 efflux in cells mimicking the human lung epithelium. For each study, additional details on culture conditions and used transporter substrates and inhibitors are provided. N/A stands for non-available

Cell type	Study type	Culture condition	Culture time	Culture format	Probe substrate	Inhibitor	Outcome	Reference
16HBE14o-	Cellular accumulation study	Liquid-coverage	4 d	Well plate	CF	MK-571	Intracellular retention increased by 12.5-fold in presence of inhibitor	[67]
Calu-3	Cellular accumulation study	Liquid-coverage	4 d	Well plate	Calcein	Probenecid, Indomethacin	Intracellular retention increased by ~ twofold in presence of inhibitors	[68]
Calu-3	Bidirectional transport	ALI	21 d	Inserts	[^3^H]-estrone-3-sulphate	MK-571	UR ~ 5, in presence of inhibitor reduced to an UR ~ 2	[42]
EpiAirway™	Bidirectional transport	ALI	21 d	Inserts	[^3^H]-estrone-3-sulphate	MK-571	UR ~ 4, in presence of inhibitor reduced to an UR ~ 2	[42]
NCI-H441	Bidirectional transport	LLI	8 d	Inserts	5,6-CF	MK-571	UR ~ 1.5, in presence of inhibitor reduced to an UR ~ 1^a^	[46]
NCI-H441	Cellular accumulation study	Liquid-coverage	7 d	Well plate	[^11^C]-MPG	MK-571	Intracellular retention of increased by > twofold in presence of inhibitor after 120 min	[73]
A549	Cellular accumulation study	Liquid-coverage^b^	N/A^b^	N/A^b^	Calcein	Probenecid	Intracellular retention increased by ~ 1.5-fold in presence of inhibitor	[74]
A549	Cellular accumulation study	Liquid-coverage	7—14 d^c^	Chamber coverslip	5,6-CDF	MK-571	Intracellular retention increased by ~ 1.4-fold in presence of inhibitor	[75]
hAELVi	Bidirectional transport	ALI	21 d	Inserts	[^3^H]-estrone-3-sulphate	MK-571	UR ~ 1.2, in presence of inhibitor reduced to an UR ~ 0.6	[61]
EpiAlveolar™	Bidirectional transport	ALI	N/A^d^	Inserts	[^3^H]-estrone-3-sulphate	MK-571	UR ~ 1.4, in presence of inhibitor reduced to an UR ~ 0.7	[61]
hAEpC	Bidirectional transport	ALI	8 d	Inserts	5,6-CF	MK-571	UR > 2, in presence of inhibitor reduced to an UR ~ 1.1	[46]

^a^The authors reported in a follow-up study that 5,6-CF is a rather suboptimal substrate for assessing MRP1-mediated efflux [72]

^b^No details on cell culture practise was found in the material and methods section [74]

^c^No concrete details for culture time of cells used for fluorophore studies [75]

^d^ Exact culture time not defined [61]

## MDR1

The expression and functional activity of MDR1 in the human lung epithelium has been extensively investigated in recent decades [2, 3]. MDR1 is known to affect the pharmacokinetics of its substrates in the human body by limiting intestinal absorption and distribution to tissues such as the brain, as well as enhancing renal and hepatobiliary clearance [4, 5]. In the lung, the picture is less clear. MDR1 was detected by various experimental techniques in several cell models of the upper and lower human lung epithelium. In general, the expression level of MDR1 in the lung epithelium appears to be lower than in other MDR1-expressing tissues such as intestine or liver. MDR1 is postulated to be expressed at the apical side of the human lung epithelium (ELF-facing side) [2].

In terms of regional expression in the lungs, MDR1 expression has been reported in cell lines derived from the upper and lower human airways including 16HBE14o-, Calu-3, NHBE, NCI-H441, A549 and hAEpCs [2]. In contrast, MDR1 appears to be absent in hAELVi cells, an alveolar cell line [61].

MDR1 showed functional activity in the majority of cells from upper and lower airways. Nevertheless, it needs to be noted that the combinational use of non-selective substrates and inhibitors for MDR1 (such as Rhodamine 123 and verapamil) impedes a clear read-out of its contributed efflux, especially in cells that express a variety of different ABC and SLC transporter. Table 2 provides an overview of in vitro studies that were conducted to investigate MDR1-mediated efflux in cells mimicking the human lung epithelium.

It is important to emphasise that we focussed within this review on studies that included a functionality assessment of expressed MDR1, whereas there are many more studies that investigated only expression levels of MDR1. There, it was shown that MDR1 expression levels are not always consistent across the same cell type, i.e. MDR1 protein was not detected by a proteomics-based mass-spectrometric readout in Calu-3 and NCI-H441 cells, whereas further studies could detect MDR1 protein in these cell lines when using techniques like Western Blot or immunofluorescent staining [31, 48, 49].

Moreover, the translation of measured in vitro MDR1 efflux in cell models to its potential impact on clinical pulmonary exposure in humans in still an open question. In terms of rodents, a few number of ex vivo studies with isolated perfused lungs hinted to the presence of active MDR1 in the pulmonary system [50, 51].

## Upper airways

The following section provides a summary of reports that assessed MDR1 efflux in cells mimicking the upper airways epithelial (i.e. 16HBE14o-, Calu-3 and primary bronchial cells). In the majority of conducted studies, the presence of efflux mediated by MDR1 was reported; however, the predominant use of non-selective substrates in combination non-selective inhibitors impedes a clear readout on the actual contribution of MDR1.

16HBE14o-, a bronchial epithelial cell line, cultured for 1 week at LLI, was assessed for the functional activity of MDR1 using Rhodamine 123 (Rh123) as MDR1-substrate in absence and presence of inhibitor (verapamil). Without inhibitor, the Rh123 secretory permeability was almost threefold higher than absorptive, indicating active mediated efflux at the apical side (ER ~ 3). In presence of verapamil, Rh123 secretory permeability decreased threefold whereas absorptive permeability did not change, with resulting ER of 1.1, confirming MDR1 functionality in 16HBE14o- cells [52]. However, both Rh123 and verapamil are not selective for MDR1 and are described to interact with other transporter proteins such as organic cation transporters (OCT) that were also described to be expressed in 16HBE14o- cells [53–55].

Calu-3, another bronchial cell line, showed a stronger expression of MDR1 at protein level when cultured at ALI for 21 days compared to cells cultured for only 8 days. This seems to be reflected in the functional efflux mediated by MDR1 in Calu-3. A bidirectional transport study compared the flux of Rh123 in absence and presence of MDR1-inhibitor PSC833 in Calu-3 cells cultured for either 8 or 21 days. Interestingly, there was no asymmetry in Rh123 transport in Calu-3 cells after 8 days and resulting permeability values did not change in presence of PSC833, suggesting the absence of active MDR1 efflux. In contrast, after 21 days, the secretory permeability was sevenfold higher than absorptive, and the net flux ratio was reduced to 2.8 in presence of PSC833. This suggests the presence of active MDR1 efflux in Calu-3 cells when cultured at ALI for 21 days [42].

Another study investigated the bidirectional flux of [^3^H]-digoxin across Calu-3 cells cultured at ALI for 21 days. Moreover, this study compared the flux in Calu-3 cells of low passage (#25–30) and high passage (#45–50). Interestingly, the asymmetry in transport of [^3^H]-digoxin is more pronounced for the low passage cells than for the high passage, whereas at protein level, the Western Blot appeared to show a more intense MDR1 band for the high passage cells (NB, no quantitative protein normalisation done). An ER of 11.4 was reported for low passage and an ER of 3.0 for the high passage. In presence of PSC833, absorptive permeability significantly increased and secretory significantly decreased, resulting in a net flux close to 1 for both passage (ER of ~ 1.2–1.4) This suggests active efflux of [^3^H]-digoxin in Calu-3 mediated by MDR1. However, also the presence of MK-571 (MRP inhibitor) was shown to have a significant impact on [^3^H]-digoxin permeability by increasing secretory and decreasing absorptive permeability. Thus, it cannot be excluded that MRPs are involved in mediating the transport of [^3^H]-digoxin across the Calu-3 cells. In the presence of sodium azide, that leads to depletion of ATP, Calu-3 cells still showed asymmetry in transport of [^3^H]-digoxin. Therefore, it was concluded that other ATP-independent transporters might be involved in [^3^H]-digoxin flux across Calu-3 cells. This emphasises the difficulty of finding a transporter probe substrate that is selective for one transporter protein and the need for careful characterisation of transporters’ activity in the used in vitro model [49].

A further study analysed the bidirectional flux of Rh123 across polarised Calu-3 cells at ALI in absence and presence of cyclosporine A. The absorptive permeability of Rh123 is around tenfold higher than secretory, and the net flux is sensitive to cyclosporine A, whose presence results in an ER < 1 [56]. However, cyclosporine A is not selective for MDR1 and was described to inhibit other ABC transporters including MRPs and BCRP, as well as SLC transporters such as OCT1 and OATPs [57–59]. Due to the broad-spectrum inhibitor properties of cyclosporine A, we recommend avoiding the use of this inhibitor when aiming to assess the contribution of individual drug transporters in cells that are known to express a variety of different ABC, SLC and SLCO transporters (such as Calu-3 [40]).

Another study investigated the bidirectional transport of Rh123 in Calu-3 cells cultured at ALI for 21 days. Rh123 showed a 2.4-fold higher secretory permeability compared to absorptive permeability (i.e. ER = 2.4), which was abolished in presence of verapamil, confirming active MDR1 efflux in Calu-3 [25].

Normal human bronchial epithelial cells were characterised for MDR1 expression by qPCR, gene microarray or mass-spectrometry-based proteomics. The results showed either weak signal for MDR1 or suggested the absence of this transporter at both gene and protein level [40, 41, 48]. Nevertheless, several in vitro studies were conducted to assess MDR1-mediated efflux in NHBE cells. One study analysed the flux of [^3^H]-digoxin across NHBE cells that were cultured at ALI for 21 days. Two cell batches of NHBE cells from the same donor were compared. Interestingly, for one batch, an ER of 1.0 was determined and for the other batch an ER of 2.3 for [^3^H]-digoxin. The authors reported that the secretory permeability of mannitol (marker of cell monolayer integrity) differed significantly between the two batches, which might explain the seen difference in secretory transport of digoxin in two cell batches from the same donor. Due to a negligible amount of MDR1 expression at gene level in NHBE cells and the inconsistent results in digoxin bidirectional transport, the authors decided to perform no bidirectional studies in presence of inhibitor [49].

A further study aimed to investigate the functional expression of MDR1 in NHBE after showing a strong signal for MDR1 at gene level by qPCR. The bidirectional flux of Rh123 was assessed after cells were cultured for 6 to 8 days at ALI. For absorptive permeability, 2.8 nm/s was reported, and 8.3 nm/s was reported for secretory permeability, resulting in an ER of ~ 3.0. In presence of verapamil to inhibit MDR1, absorptive permeability slightly increased to 4.8 nm/s and secretory slightly decreased to 7.9 nm/s, thus resulting in an ER of 1.6 with inhibitor. In general, it needs to be noted that reported permeability values in the study are relatively low and subject to higher uncertainty. Further, the study included fexofenadine (claimed MDR1 substrate) to assess its interaction with MDR1 in a bidirectional transport experiment. Fexofenadine did not show any asymmetry in transport across the NHBE cell layer with a reported *P*_app_ value of 2.8 nm/s for both absorptive and secretory permeability. The authors suggested that potential saturation of MDR1 in NHBE (as fexofenadine was tested at 500 µg/mL) could explain the lack of active transport [27].

The EpiAirway™ model is a commercially available in vitro model of the human bronchial epithelium consisting of isolated NHBE cells. It was also assessed for the expression and functional activity of MDR1 (five donors incorporated). At both gene and protein level assessed by RT-qPCR and Western Blot, MDR1 was not detected but the investigators included a bidirectional permeability study using Rh123 and PSC833. No asymmetry in Rh123 transport across the NHBE cells was measured and the presence of MDR1 inhibitor did not affect the Rh123 permeability coefficients, thus indicating the absence of MDR1 in these cells [42].

## Lower airways

In this subsection, an overview will be provided on in vitro studies that examined MDR1 efflux in cells derived from the lower human lung epithelium and included NCI-H441, A549, hAELVi or primary alveolar epithelial cells. Across all studies, Rh123 was used as substrate, and the presence of MDR1 efflux was reported in NCI-H441, A549 and the primary alveolar cells, whereas no efflux was observed in hAELVi cells and the EpiAlveolar™ model.

NCI-H441 cells, a bronchiolar cell line cultured for at least 8 days at LLI on Transwells®, was analysed for the functional activity of MDR1. Rh123 secretory permeability was 3.6-fold higher than absorptive. In the presence of LY335979 (zosuquidar, MDR1 inhibitor), absorptive permeability increased and secretory permeability decreased, resulting in a net flux of around 1, confirming functional expression of MDR1 in these cells [31].

A549 cells, an alveolar cell line, were analysed for the functional expression of MDR1 by comparing the intracellular accumulation of Rh123 in absence and presence of cyclosporine A and verapamil. The cells were cultured for 5 days in cell culture plates. The intracellular concentration of Rh123 decreased by more than 50% within 30 min after the initial loading of the cells with Rh123, thus suggesting active efflux of Rh123, likely mediated by MDR1. In the presence of either verapamil or cyclosporine A, the intracellular concentration did not change significantly, indicating the inhibition of active efflux. This data supports the finding of active mediated efflux of MDR1 in A549 cells [60]. As A549 cells do not form tight cellular barriers when cultured on inserts plates, it is not advisable to perform bidirectional efflux studies [16].

hAELVi cells cultured at ALI for 21 days did not show expression of MDR1 at protein level and only a negligible gene expression. A conducted bidirectional transport study of Rh123 did not show any asymmetry in permeability, confirming the absence of functional active MDR1 in these cells [61]. Arlo cells were not yet characterised for MDR1 efflux in a bidirectional transport study, but data from RNA bulk sequencing indicated the absence of MDR1 [38].

The expression and activity of MDR1 in the EpiAlveolar™ model were assessed in direct comparison to the hAELVi cells [61]. The EpiAlveolar™ model is a commercially available co-culture model of the human alveolar airways including primary alveolar epithelial cells, endothelial cells and fibroblasts, cultured at ALI [62]. As seen for the hAELVi cells, no asymmetry in transport of Rh123 was detected, and the addition of PSC-833 as inhibitor did not lead to any change in permeability. This result was to be expected, as MDR1 was not detected at all at protein level in this cell model [61].

A further study investigated the expression and functional activity of MDR1 in primary alveolar epithelial cells. Alveolar type II cells were isolated from lung tissue and cultured for 8 days on Transwells® where ~ 90% of the cells underwent transformation into a type I-like phenotype. Interestingly, a similar expression of MDR1 at gene level was shown (cells at day 0 vs. day 8), whereas at protein level assessed by Western Blot, a positive signal was only found for type I-like cells. To investigate the functional activity of MDR1 in the hAEpC cells, the transport of Rh123 across the cells was investigated. The secretory permeability of Rhodamine 123 was 3.1-fold higher than the absorptive permeability. In the presence of verapamil, secretory permeability decreased by 55% and absorptive increased by 92%. This data suggests the functional expression of MDR1 in the alveolar type-I like cells at the apical side [63].

## MRP1

MRP1 shows high expression levels at gene and protein level in both upper and lower airway derived cells. This includes cell lines and primary cells such as 16HBE14o-, Calu-3, NHBE, NCI-H441, A549, hAEpC, hAELVi and ARLO cells [2, 38, 61]. In comparison to other expressed members of the MRP protein family (such as MRP3/4/5/6/8), MRP1 appears to show the highest expression levels in the human lung epithelium, as shown by proteomic mass spectrometry [2, 43]. Thus, this review will focus on studies assessing the functional activity of MRP1. However, the potential interplay of other MRPs cannot be excluded. In contrast to MDR1 or BCRP, it is hypothesised that MRP1 appears to be rather expressed at the basolateral membrane of human pulmonary epithelial cells (endothelium-facing) than at the apical side (ELF-facing) [2].

MRP1 showed functional efflux in most of the investigated pulmonary cell models. However, the use of fluorophores like carboxyfluorescein or non-specific substrates such as [^3^H]-estrone-3-sulphate obstructs a clear read-out on the contribution of MRP1 efflux in lung epithelial cells. Table 3 provides an overview of in vitro studies that were conducted to investigate MRP1-mediated efflux in cells mimicking the human lung epithelium.

The potential role of MRP1 in terms of affecting pulmonary exposure of clinical drugs in humans is unknown. So far, MRP1 is not considered relevant by health authorities in terms of investigation from a drug-drug-interaction point of view, as there is no evidence for clinically relevant interactions with this protein [64]. In other tissues of the human body, it is rather MRP2 that is known to interact with drugs in the liver by actively secreting them into bile in and enhancing their elimination [65].

It was described that MRP1 might rather play an important role in transporting endogenous substrates such as glutathione and glutathione-conjugates of phase-2 metabolism and thus contributes to cells’ protection against oxidative stress [66].

## Upper airways

In the following, the findings from in vitro studies that aimed to assess the presence of MRP1 efflux in 16HBE14o-, Calu-3 and primary bronchial epithelial cells are summarised. Across all studies, a positive readout in terms of observed MRP1 efflux was reported.

To date, there are no published studies where 16HBE14o- cells have been evaluated to assess the functional activity of MRP1 in a bidirectional transport study. However, in plated format, 16HBE14o- cells were cultured for 4 days to measure the intracellular accumulation of fluorescent MRP1-substrate carboxyfluorescein (CF) in the absence and presence of MRP-inhibitor MK-571. Fluorescent CF is intracellularly formed by hydrolysis of CF-diacetate. In the presence of inhibitor, the retention of CF within the cells was increased by a factor of around 12-fold, hinting to the presence of active efflux by MRP1 in this cell line [67].

To assess the activity of MRP1 in Calu-3, Hamilton and colleagues used Calcein-AM as substrate, which is cleaved to fluorescent Calcein by intracellular ester hydrolysis. The cells were cultured at ALI for 13 to 15 days on Transwells®. The authors reported that the net secretion flux of Calcein was increased by 3.5-fold in presence of probenecid and by a factor of 2.4-fold in presence of indomethacin (MRP1 inhibitors). The individual Papp values were not reported, and the authors stated that there was a variability up to two log units in permeability between different experiments repetitions; thus, the data should be taken with caution. The same study assessed the intracellular accumulation of Calcein in Calu-3 cells that were cultured for 4 days on well plates in absence and presence of probenecid and indomethacin. Cellular retention of Calcein was increased around twofold in presence of inhibitors, hinting to functional MRP1 activity [68].

Another bidirectional study analysed the efflux of [^3^H]-estrone-3-sulphate by MRP1 in Calu-3 cells that were cultured for 21 days at ALI. The absorptive permeability was around fivefold higher than the secretory one, which indicates an efflux at the basolateral side. Interestingly, the addition of MK-571 led to a significant decrease in absorptive permeability but had no impact on secretory permeability. Thus, asymmetry in transport remained by a factor of 2, despite the presence of inhibitor [42]. Besides, it needs to be mentioned that estrone-sulphates are reported to be substrates of another ABC transporter, namely BCRP [69]. BCRP was shown to be absent in Calu-3 cells in the previous study ([42]), thus an interaction between the two ABC transporters in terms of estrone-sulphate’s efflux can be excluded. However, estrone-sulphates were described to be also transporter by organic anion transporting polypeptides transporters (OATP), and MK-571 was shown to inhibit OATPs, including OATP1B1 that was found to be expressed in the human lung epithelium [2, 70, 71]. Calu-3 cells were shown to express a variety of OATP transporters at gene levels including OATP1B1, whereas the proteins could not be detected in a LC–MS/MS-based quantification study [40, 48]. Thus, a potential interaction of [^3^H]-estrone-3-sulphate and MK-571 with OATPs in Calu-3 cells can most likely be excluded. However, this emphasises the importance of choosing a suitable substrate and inhibitor when investigating single drug transporters’ activity.

The same study investigated the functional expression of MRP1 in the EpiAirway™ model in comparison to Calu-3. The cells were cultured for 21 days at ALI. The efflux of [^3^H]-estrone-3-sulphate shows a similar behaviour to Calu-3. The absorptive permeability was around fourfold higher than the absorptive one. This ratio reduces by a factor of ~ 2 in presence of MK-571. Thus, it was concluded that MRP1 is expressed in a functional active manner at the basolateral side [42].

## Lower airways

The following subchapter provides an overview of in vitro studies that assessed MRP1 efflux in NCI-H441, A549, hAELVi and primary alveolar cells. The presence of MRP1 efflux was shown in NCI-H441, A549 and primary alveolar cells, whereas no asymmetry in transport was reported in hAELVi cells and the EpiAlveolar™ model.

The functional efflux of MRP1 in NCI-H441 cells that were cultured at LLI for around 8 days was assessed with 5,6-carboxyfluorescein (5,6-CF). The absorptive permeability was significantly higher than the secretory, but the difference appears to be less than by a factor of 2. The presence of inhibitor MK-571 decreased absorptive permeability by a factor of ~ 2 but had no significant impact on secretory permeability. In general, the measured permeability values are quite low (< 3 nm/s) [46]. The authors postulated in a recent study that 5,6-CF does not seem to be an appropriate probe substrate for assessing MRP1 efflux. A MRP1-knockout NCI-H441 cell line was established, but no significant difference in efflux of CF was found compared to wild type NCI-H441 cells. A potential upregulation of genes such as other MRPs or organic anion transporters (that are known to interact with 5,6-CF) due to the genetic knock-out process was excluded by qPCR. Thus, it was hypothesised that base line activity mediated by other MRPs or OATs in wild type and knockout cells might explain the lack of difference in CF efflux. In contrast to the previous study (see [46]), the cells were not cultured on membrane inserts but in cell culture plates. Another MRP1 substrate was tested in this study: S-(6-(7-[^11^C]-methylpurinyl))-glutathione ([^11^C]-MPG) that is intracellularly formed from its prodrug 6-bromo-7-[^11^C]-methylpurine ([^11^C]-BMP). This compound showed a significant decrease of [^11^C]-MPG efflux in MRP1-knock out vs. wild type NCI-H441 cells [72]. In a further study that used NCI-H441 cells, [^11^C]-MPG showed a significantly higher intracellular concentration in presence of MK-571 compared to non-inhibitor treated cells, hinting to the presence of active MRP1 in these cells [73].

A549 cells were shown to have a 51% increase in uptake of fluorescent Calcein-AM in presence of probenecid compared to A549 cells incubated with Calcein-AM in the absence of MRP1 inhibitor. This suggests the presence of active efflux of MRP1 in A549 cells [74]. A further study in A549 came to a similar result where the intracellular concentration of 5,6-Carboxy-2′,7′-dichlorofluorescein (5,6-CDF) was increased by ~ 40% in presence of inhibitor MK-571. As it was postulated that MRP1 transporter activity is related to the presence of glutathione, the same study investigated the intracellular concentration of 5,6-CDF in presence of N-acetylcysteine (precursor molecule for glutathione) or in presence of buthionine sulfoximine (inhibitor of glutathione synthesis). Interestingly, the intracellular concentration of 5,6-CDF increased by more than twofold in presence of buthionine sulfoximine (likely due to lowered MRP1 activity) and decreased in by ~ 40% in presence of N-acetylcysteine (likely due to increased MRP1 activity) [75].

hAELVi cells cultured at ALI for 21 days were assessed for MRP1 by comparing the absorptive and secretory permeability of [^3^H]-estrone-3-sulphate in presence and absence of MK-571. Interestingly, there was no significant difference between absorptive and secretory permeability, resulting in an UR of ~ 1. In presence of MK-571, the UR decreased to ~ 0.5. Due to the impact of inhibitor on UR, the authors concluded a functional activity of MRP1 at the basolateral side of hAELVi cells [61]. However, it needs to be kept in mind that there is no asymmetry in transport without inhibitor, which is questioning the presence of active MRP1 efflux.

Arlo cells were not yet characterised for MRP1 activity, but RNA-sequencing hinted to presence of MRP1 [38].

MRP1 activity has also been assessed in the EpiAlveolar™ model. In absence of an inhibitor, [^3^H]-estrone-3-sulphate showed an UR of ~ 1.3, which reduced to ~ 0.7 in presence of inhibitor MK-571. The authors concluded a functional efflux at the basolateral side [61]. As there was no asymmetry in permeability of [^3^H]-estrone-3-sulphate spotted in absence of inhibitor, the presence of MRP1 efflux is questionable and conducting the study with a further MRP1 substrates would have been beneficial.

Another study investigated hAEpC (differentiated from isolated type II alveolar cells) in terms of MRP1 efflux. 5,6-CF was used as substrate and absorptive permeability is more than twofold higher than secretory. The presence of a MRP1 inhibitor (MK-571) led to a significant decrease of absorptive permeability and increase of secretory, resulting in an UR of ~ 1.1. Thus, the authors concluded that 5,6-CF is a substrate of active efflux by MRP1 at the basolateral membrane [46].

## BCRP

BCRP expression has been detected at gene and protein level in cell lines and primary cells of upper and lower airways. This includes 16HBE14o-, Calu-3, NCI-H441, A549, NHBE and hAEpC cells [2]. In hAELVi cells, BCRP appears to be absent, and RNA sequencing data showed a rather weak expression in Arlo cells [38, 61]. The expression of BCRP was reported to be on the apical side of the human lung epithelium [3]. Across the reports, however, there are only a limited number of studies assessing the functional activity of BCRP.

In terms of BCRP efflux in the human lung, data hints to the absence or presence of rather weak BCRP efflux in the majority cells mimicking the human airways. This is summarised in Table 4, which provides an overview of in vitro studies that were conducted to investigate BCRP-mediated efflux in cells mimicking the human lung epithelium.

**Table 4:** Summary and outcome of in vitro studies conducted to investigate BCRP efflux in cells mimicking the human lung epithelium. For each study, additional details on culture conditions and used transporter substrates and inhibitors are provided. N/A stands for non-available

Cell type	Study type	Culture condition	Culture time	Culture format	Probe substrate	Inhibitor	Outcome	Reference
Calu-3	Cellular accumulation study^a^	Liquid-covered	11–13 d	Well plate	[^3^H]-mitoxantrone	Elacridar	2.7-fold increase in intracellular concentration in presence of inhibitor	[77]
Calu-3	Cellular accumulation study^a^	Liquid-covered	11–13 d	Well plate	Hoechst 33342	Elacridar	1.7-fold increase in intracellular concentration in presence of inhibitor	[77]
EpiAirway™	Bidirectional transport	ALI	21d	Inserts	[^3^H]-mitoxantrone	Febuxostat	ER ~ 1.3, in presence of inhibitor ER ~ 1	[42]
NCI-H441	Bidirectional transport	ALI	≥ 7 d	Inserts	BODIPY™ FL prazosin	Ko143	ER ~ 1.7, reduced in presence of inhibitor to ER ~ 1.0	[80]
A549	Cellular accumulation study	Liquid-covered	2 d	Well plate	Hoechst 33342	Fumitremorgin C	~ 1.5-fold increase of intracellular concentration in presence of inhibitor	[81]
hAELVi	Bidirectional transport	ALI	21 d	Inserts	BODIPY™ FL prazosin	Ko143	ER < 1 in absence and presence of inhibitor	[61]
EpiAlveolar™	Bidirectional transport	ALI	N/A^b^	Inserts	BODIPY™ FL prazosin	Ko143	ER ~ 1.0 in absence and presence of inhibitor	[61]
hAEpC	Bidirectional transport	ALI	7–9 d	Inserts	BODIPY™ FL prazosin	Ko143	UR ~ 1.8, that was still present in presence of inhibitor^c^	[80]

^a^Further BCRP inhibitors were tested in this study [77]

^b^Exact culture time not defined [61]

^c^The authors claimed that another active transport system might be responsible for the seen asymmetry in transport [80]

In contrast of MDR1 and MRP1, there were less in vitro studies that aimed to elucidate the role of BCRP-mediated efflux at the human lung epithelium. This is interesting because BCRP is known (as MDR1) to affect the pharmacokinetics of its substrates in a clinical-relevant manner, e.g. by limiting intestinal absorption, which can result in drug-drug interactions [76]. Thus, it is also required by health authorities such as the FDA to test new compounds for their interaction with human BCRP [64]. Nevertheless, its potential role in driving human pulmonary pharmacokinetics requires further research.

## Upper airways

This section will provide an overview of in vitro studies that assessed BCRP efflux in Calu-3 (by cellular accumulation studies) and in primary bronchial epithelial cells (EpiAirway™ model) by bidirectional transporter studies, whereas only for Calu-3, the presence of active BCRP efflux was reported.

To our knowledge, there are no in vitro studies available that have evaluated BCRP-mediated efflux in 16HBE14o- cells.

In terms of BCRP efflux in Calu-3 cells, no bidirectional transporter assessment has been published to date. A study by Paturi and colleagues aimed to investigate BCRP-mediated efflux in Calu-3 at LLI by measuring the intracellular concentration of [^3^H]-mitoxantrone and Hoechst 33342 (BCRP substrates) in the absence and presence of BCRP inhibitors such as elacridar. In the presence of elacridar, intracellular concentration of [^3^H]-mitoxantrone increased up to a maximum 2.67-times in a concentration-dependent manner of inhibitor (0.1 to 200 µM). Hoechst 33342 intracellular concentration also increased up to 1.68-fold in presence of elacridar [77]. However, it needs to be noted that elacridar also inhibits MDR1 and that mitoxantrone as well as Hoechst 33342 have been shown to be a substrate of human MDR1 in vitro. Since MDR1 was described to be expressed in Calu-3, a potential interaction of chosen substrates and inhibitor with MDR1 cannot be excluded [2, 45, 78, 79]. This emphasises that choosing a selective substrate and inhibitor for ABC efflux studies can be challenging and shows the importance of careful interpretation of these studies.

Immunostaining pointed to an expression of BCRP at both apical and basolateral side of the membrane of the EpiAirway™ model (cultured at ALI). This is reflected by the absence of asymmetry in absorptive and secretory permeability of BCRP substrate [^3^H]-mitoxantrone (resulting ER of ~ 1). The addition of febuxostat (BCRP inhibitor) only had a minor effect on measured permeability coefficients [61].

## Lower airways

In this section, in vitro studies were summarised that included an assessment of BCRP efflux in either NCI-H441, A549, hAELVi or hAEpC cells. Overall, a weak or the complete absence of BCRP efflux was reported.

NCI-H441 cells have been shown to express similar levels of BCRP at protein level when cultured at ALI vs. LLI for 1 week after analysis by Western Blot. The same study assessed the functional activity of BCRP via a bidirectional transport study using BODIPY™ FL prazosin. Secretory permeability was significantly higher than absorptive, around 1.7-fold. Interestingly, the addition of Ko143 as inhibitor led to an increase of prazosin permeability in the absorptive direction, whereas secretory permeability was not changed significantly, resulting in an ER of 1.0 [80]. The data hints to a weak BCRP-mediated efflux in the NCI-H441 cell line.

A549 cells were shown to express functionally active BCRP as the intracellular accumulation of Hoechst 33342 increased by around 50% in presence of BCRP inhibitor Fumitremorgin C. In this study, the cells were seeded onto 96-well culture plates for 48 h [81].

hAELVi cells at ALI were analysed for their functional expression of BCRP by bidirectional transport studies of BODIPY™ FL prazosin with and without Ko143 or febuxostat as BCRP inhibitors. The derived apparent permeability values were all similar to each other, and addition of inhibitors had no impact on permeability coefficients. The absence of BCRP-mediated efflux was expected as BCRP was only weakly expressed at gene level and absent at protein level. The same observation was made for the EpiAlveolar™ model, where the BCRP protein was not detected by Western Blot and bidirectional efflux studies did not observe any asymmetry in transport of prazosin [61].

The bidirectional flux of BODIPY™ FL prazosin was assessed across hAEpC cells that were derived from isolated type II alveolar epithelial cells cultured on Transwell® inserts for at least 1 week. Interestingly, absorptive permeability was twofold higher than secretory permeability, which hints to a transporter acting at the basolateral side. In the presence of Ko143 as inhibitor of BCRP, a net absorption of around twofold remained. Therefore, the authors suggested that a transporter protein other than BCRP might explain the net absorption of prazosin in the hAEpC cells. The study also showed that BCRP expression decreases when AT-2 cells differentiate into AT-1-like cells [80].

## Behind the data: factors that shape transporter study outcomes

**Fig. 5:**
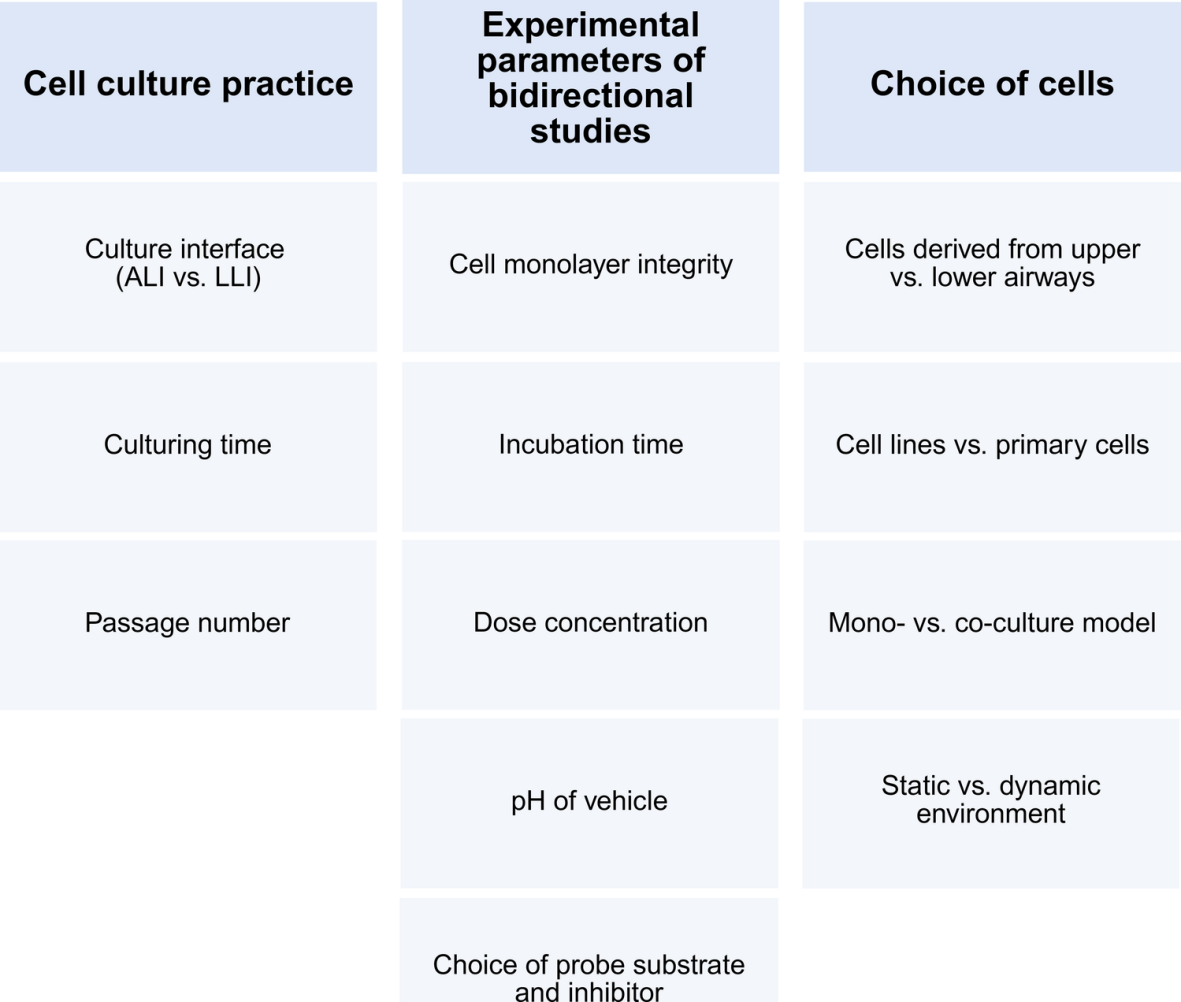
Overview of factors that are import to consider when conducting and interpreting pulmonary bidirectional permeation and transporter studies. Image created in https://BioRender.com

In vitro models can be used to assess pulmonary permeation and investigate drug transporter-mediated processes in a relatively medium to high-throughput manner, if screening uses cell line-based Transwell® models. However, there are still certain factors to consider when conducting transport and permeability studies and when interpreting data from these kinds of experiments. Figure 5 illustrates a number of different points that need to be carefully assessed.

### Cell culture conditions

Different and non-standardised culture conditions, such as ALI vs. LLI culture, have been reported to impact the morphological development of cells like Calu-3. It was shown that Calu-3 cells at ALI differentiate into a more native-like epithelium described as ‘pseudostratified-like’ with numerous microvilli and secretory vesicle, that contain glycoproteins, resulting in an enhanced mucus secretion, whereas Calu-3 were differentiating into a simple-layered ‘cuboidal-like’ epithelium, with a threefold lower average height and less secretory vesicle when cultured at LLI. Moreover, it was revealed that the culture interface affects the formation of tight junctional protein ZO-1, which was reduced when Calu-3 cells were cultured at ALI, resulting in threefold lower TEER values compared to LLI [24, 82, 83].

Further studies investigated the impact of culture conditions on ABC transporter’s expression and efflux. One study compared MDR1-mediated transport in Calu-3 at ALI after 7 and 21 days culturing on Transwell® inserts and demonstrated that no active transport was present after 7 days, whereas after 21 days, the probe substrate Rh123 shows significant higher secretory than absorptive permeability (~ sevenfold) [42]. This emphasises the need of a certain culture time for Calu-3 to polarise ABC transporter expression and activity (similar to Caco-2 cells, a human colon cancer epithelial cells, which is typically cultured for 21 days on Transwell® in order to express ABC transporter in a polarised manner [84]). Moreover, the culture interface (ALI vs. LLI) was described to influence the expression level of certain ABC and SLC by gene up- or downregulation (readout via qPCR). More studies would need to assess how this translates into protein expression and transporter functionality [24]. In a further study, the effect of cell passage number on MDR1-mediated efflux in Calu-3 was investigated. The cells were shown to have ~ fourfold lower MDR1 efflux of Rhodamine 123 at higher passage number (≥ 45) compared to lower passages (≤ 30) [49].

If all these potential confounding factors (such as culture interface, cell passage number and time of culturing) can affect ABC transporter expression and resulting functional efflux in further cells, it requires more research. Nevertheless, these examples in Calu-3 emphasise the need for a careful characterisation of in vitro systems before conducting transporter experiments and evaluate the experimental setting closely when comparing findings to other studies.

### Experimental parameters of bidirectional studies

In addition to culture conditions, the experimental design of the conducted transporter functionality studies can affect the outcome.

First, in order to be a suitable model to assess drug transport and permeation processes in vitro, the cells need to form a tight and confluent layer. The integrity of the cell layer needs to be granted, so that compounds can reach the receiver chamber only by permeating across the cells. The tightness of this barrier is achieved by formation of tight junctions, which restrict paracellular transport [85, 86]. It is recommended to do a quantitative assessment by measuring the paracellular permeability of a hydrophilic marker such as [^14^C]-mannitol or Lucifer yellow. In this context, TEER measurement was described to be another quality control and can be used to assess cellular tightness before and after an experiment in addition to paracellular marker experiments [44].

Second, it is important to determine an optimal incubation time for permeability assessments. Transcellular studies are usually conducted by dosing the compound of interest into the apical compartment and taking samples from basolateral compartment (in this case, the receiver chamber) and vice versa. The *P*_app_ is a common read-out in these studies and describes the compound movement from the donor into the receiver over time. This process into the receiver chamber is time-proportional; typical permeability profiles comprise an initial lag phase, followed by a linear phase and final lag phase when steady state is reached. It is recommended to perform the sampling within the linear phase in order to retrieve a representative and accurate permeability estimate. Compounds that have a lower passive permeability, e.g. due to being highly lipophilic (log*P* ≥ 3), need a longer time to reach linear phase. Thus, a short incubation time bears the risk of underestimating the compound’s permeability (i.e. measuring within initial lag phase). It was simulated in a three compartment model that compounds with an apparent permeability lower than 28 nm/s need longer than 120 min to reach the linear phase. Transcellular permeability studies across Caco-2 cells mainly confirmed the fitting of the mathematical model. However, if the incubation time is too long and sink condition cannot be guaranteed any more, there is a risk for backflux of compound from receiver to donor and resulting misestimation of the apparent permeability [87].

Furthermore, it is important to choose a suitable substrate dosing concentration, when investigating the role of drug transporters. A high incubation concentration of the substrate might saturate the transport mechanism of interest and abolish the transporter-mediated activity. Usually the Michaelis–Menten constant (*K*_m_), which describes the substrate concentration at which the transport rate is at half of the maximum is used as parameter to assess saturation concentration and define an appropriate dose concentration [88]. A study, which measured the flux of moxifloxacin (fluoroquinolone antibiotic) across a Calu-3 monolayer in order to investigate its interaction with MDR1, dosed moxifloxacin at a concentration of 50 µM. Initial experiments showed that the *K*_m_ of moxifloxacin against MDR1 from basolateral to apical direction in Calu-3 is 148 µM. Thus, 50 µM was chosen as suitable dose concentration, since MDR1-mediated transport should not be saturated at this dose [89]. However, it is not precluded that other potentially involved drug transporters might be saturated at this dose.

The calculation of the mass balance recovery, which assesses the sum amount of test compound in donor and acceptor compartment at the end of the incubation relative to initial dose concentration, allows an assessment of the fraction of drug that might be ‘lost’ in the cell during the incubation period, e.g. due to lysosomal trapping, binding to acidic phospholipids or nonspecific binding to the plastic ware. It has been estimated that for each 10% reduction in recovery, *P*_app_ is underestimated by approximately 22 nm/s, mainly in the context of lysosomal trapping. The understanding of the factors of poor recovery can contribute to minimise the risk of a compound’s permeability underestimation [90]. It might be possible that recovery values for the same drug are different across various cells (e.g. due to cell-specific metabolic processes). Rarely, recovery values are reported in literature and thus impede a clear interpretation of received permeability results.

It has also been shown that the pH value of the vehicle in which the drug is dissolved during transcellular studies can have an impact on permeability. The apical to basolateral permeability of salbutamol and formoterol was compared at apical pH values ranging from 6 to 8. The permeability of salbutamol was not affected by any change in pH. In contrast, the absorptive permeability of formoterol increases from 12.6 to 39.8 nm/s with increasing pH. The reason for this is the increasing proportion of unionised species of formoterol with elevated pH and thus higher membrane permeation compared to the ionised species [86, 91]. Typically, solutions such as Hank’s buffered salt solution or Krebs buffer solution that are adjusted to a pH of 7.4 (physiological blood pH) are used for transcellular studies [63, 89]. This is reasonable when assessing transport processes from the basolateral to apical side (mimicking blood to ELF side). However, the pH in airways is described to be lower (~ 6.0 to 7.0) than in blood [92]. Thus, when assessing the permeability of an ionisable compounds from apical to basolateral site (ELF to blood site), a pH adjustment of the liquid in apical site may be reasonable.

Another critical consideration when performing bidirectional transporter studies is the choice of specific substrates and inhibitors. This is emphasised for MDR1, which is by far the most investigated efflux transporter in the lung [93]. Its functional expression is often explored by performing transport studies with Rh123 as substrate for MDR1 [42, 63]. However, it was shown that Rh123 is not only a substrate of MDR1 but also of OCT1/2 and possibly also of other MRPs [53, 94]. Digoxin is another commonly used MDR1 probe substrate [45]. Nevertheless, studies have indicated that digoxin’s drug disposition in a Calu-3 is not driven by MDR1 solely but other not yet defined transporter systems appear to be involved [49]. Verapamil is often used as an inhibitor as it is a well-known inhibitor for MDR1-mediated efflux [63, 95, 96]. However, verapamil was shown to inhibit uptake transporters such as OCTN2 [97]. OCTN2 is reported to be expressed in the lung [2]. Thus, verapamil might not be an optimal choice when assessing the role of MDR1 in pulmonary cells. Instead, zosuquidar might be used as an alternative since it is a highly selective and potent MDR1 inhibitor [98]. In addition, PSC-833 (valspodar) appears to be a selective MDR1 inhibitor and was used in several in vitro studies to understand the role of MDR1 in the human lung epithelium [42, 99, 100]. For MRP1, MK-571 appears to be a suitable inhibitor, although this compound is not specific for MRP1, but also inhibits other members of the MRP family such as MRP2 or MRP4 [101, 102]. Moreover, it was described that MK-571 has an inhibitory effects on OATPs [103]. As a substrate for pulmonary MRP1, 5(6)-CF was proposed [46]. However, a recent study revealed that this compound does not seem to be an optimal substrate for MRP1, as the release of 5(6)-CF was not significantly different between NCI-H441 wild and MRP1-knockout cells [72]. Oncologic drugs such as such vinca-alkaloids or HIV protease inhibitor were described to be substrates of MRP1, but often, these compounds are shared substrates with MDR1 [45, 104]. To assess the activity of pulmonary BCRP, prazosin and mitoxantrone have been used as substrates [42, 80]. However, both drugs were also reported to be substrates of MDR1 [45]. Ko143 is often used as an inhibitor to assess the activity of BCRP [80]. A study has found that Ko143 has the potential to also inhibit MDR1 and MRP1 at concentrations ≥ 1 µM, thus not being selective for BCRP anymore if a too high inhibitor concentration is chosen [105]. This shows that the lack of transporter-specific inhibitors and substrates is a huge challenge when characterising drug transporter expression patterns in cells, which are known to express a variety of different transporter proteins [106].

### Choice of the cells

Since bronchial and alveolar lung regions differ in morphology and structure and a difference in drug transporter expression pattern is proposed, it appears as necessary to use different cell types when assessing bronchial compared to alveolar drug disposition [2, 107]. However, this needs further investigation to explore if drug transporter activity and the resulting impact on the distribution of ABC transporter substrates are significantly different in the upper airways vs. the lower airways [16].

Another question to reflect on is the use of cell lines vs. primary cells for drug distribution studies. Cell lines are established either by viral immortalization of primary cells or by isolation from tumours. Cell lines bear the advantage of having an almost unlimited lifespan and rely on known culture techniques. This is a significant advantage in terms of regular supply and the need of future standardisation and validation to eventually gain regulatory acceptance (also as an alternative to animal testing). Moreover, compared to primary cells, which are isolated directly from human tissue, no donor-to-donor variability needs to be taken into account, and there is no need for a sometimes-complex isolation processes. Nevertheless, due to their immortalization or cancerous origin, it remains unclear to what degree a cell line is mimicking the physiological phenotype in vivo [34, 108–110]. Thus, it was proposed that readouts of transporters’ expression and functionality studies with cell lines should be compared to findings from healthy primary cells (tissue) [16]. A study quantified the expression of a panel of drug transporters at protein level using an LC–MS/MS approach in a variety of cell lines and compared it to human primary cells from trachea, bronchi and alveoli. Alignments and disconnects in transporter expression levels of were shown, e.g. when comparing primary tracheal and bronchial derived cells to Calu-3 cells (bronchial-derived cell line). As example, MDR1 could neither be detected in Calu-3 nor in primary cells of the trachea and bronchial region, whereas BCRP showed an almost 6- higher expression in Calu-3 compared to primary bronchial cells. MRP1 shows comparable expression levels in both Calu-3 and bronchial cells. However, it needs to be mentioned that all cells in this study were cultured for 5 days and Calu-3 were shown to polarise in terms of MDR1 expression after 21 days [48, 49].

Another consideration to remember is that the lung in vivo is more complex since it consists of a variety of different cell types additional to epithelial cells such as capillary endothelial cells, interstitial cells (e.g. fibroblasts) and immune cells (e.g. alveolar macrophages) [111]. Vascular endothelial cells in tissues such as the brain were shown to express many different drug transporters [112]. The human pulmonary vasculature is described to cover a surface area of around 125 m^2^ [113]. However, the expression of drug transporters in the lung vasculature is still largely unknown. Immunohistochemical staining of MDR1 in frozen tissue indicated that bronchial endothelial cells seem to express MDR1 (dependent on the antibody used for staining) and alveolar endothelial cells do not appear to express MDR1 [114]. Nevertheless, more studies are needed to assess the expression of drug transporters in the human lung vasculature and to confirm the MDR1 expression in bronchial endothelial cells [3]. Moreover, it was shown that alveolar macrophages express different drug transporters, which might influence intracellular concentration in macrophages, as well as local pulmonary drug concentration in ELF for transporter substrates [115]. In literature, lung co-cultures up to quadruple-co-culture systems are described (e.g. combining epithelial with endothelial cells with or without different immune cells) [16]. If such a co-culture system would be more beneficial than a monoculture in terms of predicting accurately human lung exposure of drugs, it remains an open question.

Over the last years, the interest in complex in vitro models such as organ-on-a-chip has grown, and these systems are often described to closely mimic the human physiological microenvironment with overcoming limitations from simple 2D cell culture systems [116]. One of the limitations of lung epithelial cells cultured on stiff porous filter membranes is the lack of mechanical stretch, whereas in physiological state, the lung is constantly exposed to strain (breathing process). Few in vitro studies assessed the impact of dynamic stretch on the pulmonary permeability of two cellular integrity markers: fluorescein isothiocyanate (FITC)-sodium (0.4 kDa) and Rhodamine B isothiocyanate (RITC)-dextran (70 kDa). One study used 16HBE14o- cells that were incorporated into a breathing lung-on-a-chip model by seeding onto the apical side of a stretchable and porous polydimethylsiloxane membrane. When the cells reached confluence after 72 h, they were either kept in static conditions or stretched for 19 h. Afterwards, the permeability of FITC-sodium and RITC-dextran was measured from apical to basolateral direction in static or dynamic environment. The transport of FITC-sodium increased by ~ 46% in the dynamic condition compared to static, whereas the transport of RITC-dextran did not change significantly. The authors hypothesised that due to the stretching the intercellular pores were stretched and thus enhancing the paracellular permeability of small hydrophilic molecules such as FITC-sodium (RITC-dextran likely not affected due to its high molecular weight of 70 kDa) [117]. In a follow-up study with an alveolus-on-chip model, the authors showed that applying cyclic stretch to hAEpC cells significantly increased both the permeability of FITC-sodium and RITC-dextran (~ sevenfold). In contrast to the study with 16HBE14o- cells, the primary hAEpC cells were longer exposed to cyclic stretch (more than threefold longer) and cultured at ALI. Another explanation could be that the hAEpC are more sensitive to mechanical stretch than the 16HBE14o- cell line. How this translates to drug absorption in the human lung is still an open question and needs further research [118]. To the best of our knowledge, we are not aware of any publication that investigated the functional activity of ABC transporters in the human lung epithelium in an advanced lung-on-a-chip model.

## Conclusion

Within the scope of this review, it was shown that ABC transporter proteins seem to be expressed in a functional active manner in cell models mimicking the human upper and lower airways. Nevertheless, differences in the expression patterns were shown, which might be impacted by cell origin (cancer cells vs. immortalised vs. primary cells) and further factors. In general, MRP1 showed efflux in majority of upper and lower airway cells, whereas MDR1 efflux appears to be mainly present in bronchial and bronchiolar cells and BCRP efflux was basically absent or weak across all cells. As already stated, the use of non-specific transporter substrates and inhibitors often impedes a clear understanding of the contribution of single transporter protein.

Moreover, we have pinpointed to important aspects that need to be considered, when conducting pulmonary permeability and transport studies in vitro. There are no clear defined protocols, thus resulting in variation in methods of different studies even if the same transporter protein is analysed within the same cell type. As shown above for Calu-3 as example (see the ‘Cell culture conditions’ section), tiny differences in cell culture and experimental set-up can influence the outcome and thus potentially resulting in conflicting data on pulmonary drug transporters’ activity. Across the summarised studies, it is also not evident when the interaction of a substrate with an ABC transporter is claimed to be positive, i.e. confirming the presence of active efflux. Often, active ABC transporter efflux is assumed to be present when there is statistical difference in absorptive and secretory permeability (Papp AB and Papp BA) of the substrate. In the context of transporter-mediated drug-drug interactions, health authorities like the FDA classify compounds as substrates of MDR1 if there is ≥ twofold differences in net flux in a bidirectional transporter in vitro study across polarised epithelial cells. Moreover, this asymmetry needs to be significantly reduced in presence of a MDR1-inhibitors [5]. These two criteria are not always met in the summarised studies of this review. There is a need to discuss if similar criteria need to be applied when assessing data of pulmonary transporter studies.

To our knowledge, there are no known clinical interactions of drugs with ABC transporters at the level of the human lung epithelium. Thus, a remaining open question is how the in vitro findings that are summarised in this review can be translated to clinical drug exposure data in humans and if ABC transporters can significantly affect local pulmonary pharmacokinetic processes of their substrates. For this, more pulmonary in vitro ABC transporter studies with drugs, for which not only human clinical plasma, but also ELF data is available, would be needed to understand if there is potential correlation between in vitro findings and clinical exposure data.

## Data Availability

Data sharing is not applicable to this article as no datasets were generated or analysed during this study.

## Abbreviations

ABC: ATP-binding cassette
ALI: Air-liquid interface
BCRP: Breast cancer resistance protein
5,6-CDF: 5,6-Carboxy-2′,7′-dichlorofluorescein
5,6-CF: 5,6-Carboxyfluorescein
CF: Carboxyfluorescein
[^11^C]-BMP: 6-Bromo-7-[^11^C]-methylpurine
[^11^C]-MPG: S-(6-(7-[11C]-methylpurinyl))-glutathione
ELF: Epithelial lining fluid
ER: Efflux ratio
FITC: Fluorescein isothiocyanate
hAEpC: Type I-like alveolar pneumocytes
*K*_m_: Michaelis-Menten constant
LLI: Liquid-liquid interface
MDR1: Multidrug resistance protein 1
MRP: Multidrug resistance associated protein
NHBE: Normal human bronchial epithelial cells
OATP: Organic anion transporting polypeptide
OCT: Organic cation transporter
*P*_app_: Apparent permeability
*P*_app_ AB: Apparent permeability from apical to basolateral side (*absorptive*)
P_app_BA: Apparent permeability from basolateral to apical side (*secretory*)
PCR: Polymerase chain reaction
Rh123: Rhodamine 123
RITC: Rhodamine B isothiocyanate
SLC: Solute carrier
SLCO: Solute carrier organic anion
TEER: Transepithelial electrical resistance
UR: Uptake ratio

## References

[1] Nigam SK. What do drug transporters really do? Nat Rev Drug Discov. 2015;14(1):29–44. 25475361 10.1038/nrd4461 PMC4750486

[2] Nickel S, et al. Transport mechanisms at the pulmonary mucosa: implications for drug delivery. Expert Opin Drug Deliv. 2016;13(5):667–90. 26909544 10.1517/17425247.2016.1140144

[3] Gumbleton M, et al. Spatial expression and functionality of drug transporters in the intact lung: objectives for further research. Adv Drug Deliv Rev. 2011;63(1–2):110–8. 20868712 10.1016/j.addr.2010.09.008

[4] Borst P, Schinkel AH. P-glycoprotein ABCB1: a major player in drug handling by mammals. J Clin Invest. 2013;123(10):4131–3. 24084745 10.1172/JCI70430 PMC3784548

[5] Giacomini KM, et al. Membrane transporters in drug development. Nat Rev Drug Discov. 2010;9(3):215–36. 20190787 10.1038/nrd3028 PMC3326076

[6] You G, Morris ME. Overview of drug transporter families. In: You G, Morris ME, editors. Drug transporters: molecular characterization and role in drug disposition. 2nd ed. New Jersey: John Wiley & Sons; 2014. p. 1–6.

[7] Sporty JL, Horalkova L, Ehrhardt C. In vitro cell culture models for the assessment of pulmonary drug disposition. Expert Opin Drug Metab Toxicol. 2008;4(4):333–45. 18433340 10.1517/17425255.4.4.333

[8] Patton JS, Byron PR. Inhaling medicines: delivering drugs to the body through the lungs. Nat Rev Drug Discov. 2007;6(1):67–74. 17195033 10.1038/nrd2153

[9] O’Donnell KP, Smyth HDC. Macro- and microstructure of the airways for drug delivery. In: Hickey AJ, editor. Controlled pulmonary drug delivery. Springer; 2011. p. 1–19.

[10] Franciosi L, et al. Proteomics of epithelial lining fluid obtained by bronchoscopic microprobe sampling. Methods Mol Biol. 2011;790:17–28. 21948403 10.1007/978-1-61779-319-6_2

[11] Crystal RG, et al. Airway epithelial cells: current concepts and challenges. Proc Am Thorac Soc. 2008;5(7):772–7. 18757316 10.1513/pats.200805-041HR PMC5820806

[12] Rokicki W, et al. The role and importance of club cells (Clara cells) in the pathogenesis of some respiratory diseases. Kardiochir Torakochirurgia Pol. 2016;13(1):26–30. 27212975 10.5114/kitp.2016.58961 PMC4860431

[13] Joshi N, Walter JM, Misharin AV. Alveolar Macrophages. Cell Immunol. 2018;330:86–90. 29370889 10.1016/j.cellimm.2018.01.005

[14] Harkema JR, Nikula KJ, Haschek WM, et al. Chapter 14 - Respiratory system. In: Wallig MA, et al., editors. Fundamentals of toxicologic pathology. 3rd ed. London: Academic Press; 2018. p. 351–93.

[15] Wang Y, et al. Pulmonary alveolar type I cell population consists of two distinct subtypes that differ in cell fate. Proc Natl Acad Sci U S A. 2018;115(10):2407–12. 29463737 10.1073/pnas.1719474115 PMC5877944

[16] Selo MA, et al. In vitro and ex vivo models in inhalation biopharmaceutical research - advances, challenges and future perspectives. Adv Drug Deliv Rev. 2021;177: 113862. 34256080 10.1016/j.addr.2021.113862

[17] Richter C, et al. Chapter 3.5 - Cell-based in vitro models for pulmonary permeability studies. In: Sarmento B, Leite Pereira C, and Das Neves J, editors. Concepts and models for drug permeability studies. 2nd ed. Cambridge: Woodhead Publishing; 2024. pp. 137–168

[18] Hiemstra PS, Tetley TD, Janes, SM. Airway and alveolar epithelial cells in culture. Eur Respir J. 2019; 10.1183/13993003.00742-2019 10.1183/13993003.00742-2019 31515398

[19] Srinivasan B, et al. TEER measurement techniques for in vitro barrier model systems. J Lab Autom. 2015;20(2):107–26. 25586998 10.1177/2211068214561025 PMC4652793

[20] Cozens AL, et al. CFTR expression and chloride secretion in polarized immortal human bronchial epithelial cells. Am J Respir Cell Mol Biol. 1994;10(1):38–47. 7507342 10.1165/ajrcmb.10.1.7507342

[21] Forbes B, et al. The human bronchial epithelial cell line 16HBE14o- as a model system of the airways for studying drug transport. Int J Pharm. 2003;257(1–2):161–7. 12711171 10.1016/s0378-5173(03)00129-7

[22] Ehrhardt C, et al. Influence of apical fluid volume on the development of functional intercellular junctions in the human epithelial cell line 16HBE14o-: implications for the use of this cell line as an in vitro model for bronchial drug absorption studies. Cell Tissue Res. 2002;308(3):391–400. 12107432 10.1007/s00441-002-0548-5

[23] Shen BQ, et al. Calu-3: a human airway epithelial cell line that shows cAMP-dependent Cl- secretion. Am J Physiol. 1994;266(5 Pt 1):L493-501. 7515578 10.1152/ajplung.1994.266.5.L493

[24] Kreft ME, et al. The characterization of the human cell line Calu-3 under different culture conditions and its use as an optimized in vitro model to investigate bronchial epithelial function. Eur J Pharm Sci. 2015;69:1–9. 25555374 10.1016/j.ejps.2014.12.017

[25] Haghi M, et al. Time- and passage-dependent characteristics of a Calu-3 respiratory epithelial cell model. Drug Dev Ind Pharm. 2010;36(10):1207–14. 20374185 10.3109/03639041003695113

[26] Martin J, et al. Characterization of a primary cellular airway model for inhalative drug delivery in comparison with the established permanent cell lines CaLu3 and RPMI 2650. In Vitro Model. 2024;3(4–6):183–203. 39872698 10.1007/s44164-024-00079-y PMC11756470

[27] Lin H, et al. Air-liquid interface (ALI) culture of human bronchial epithelial cell monolayers as an in vitro model for airway drug transport studies. J Pharm Sci. 2007;96(2):341–50. 17080426 10.1002/jps.20803

[28] Min KA, et al. Functional and cytometric examination of different human lung epithelial cell types as drug transport barriers. Arch Pharm Res. 2016;39(3):359–69. 26746641 10.1007/s12272-015-0704-6 PMC4794378

[29] Min KA, Rosania GR, Shin MC. Human airway primary epithelial cells show distinct architectures on membrane supports under different culture conditions. Cell Biochem Biophys. 2016;74(2):191–203. 26818810 10.1007/s12013-016-0719-8 PMC4903904

[30] Ren H, Birch NP, Suresh V. An optimised human cell culture model for alveolar epithelial transport. PLoS ONE. 2016;11(10): e0165225. 27780255 10.1371/journal.pone.0165225 PMC5079558

[31] Salomon JJ, et al. The cell line NCl-H441 is a useful in vitro model for transport studies of human distal lung epithelial barrier. Mol Pharm. 2014;11(3):995–1006. 24524365 10.1021/mp4006535

[32] Vuong H, et al. JNK1 and AP-1 regulate PMA-inducible squamous differentiation marker expression in Clara-like H441 cells. Am J Physiol Lung Cell Mol Physiol. 2002;282(2):L215–25. 11792626 10.1152/ajplung.00125.2001

[33] Lieber M, et al. A continuous tumor-cell line from a human lung carcinoma with properties of type II alveolar epithelial cells. Int J Cancer. 1976;17(1):62–70. 175022 10.1002/ijc.2910170110

[34] Haghi M, et al. Across the pulmonary epithelial barrier: integration of physicochemical properties and human cell models to study pulmonary drug formulations. Pharmacol Ther. 2014;144(3):235–52. 24836727 10.1016/j.pharmthera.2014.05.003

[35] Barosova H, et al. Inter-laboratory variability of A549 epithelial cells grown under submerged and air-liquid interface conditions*.* Toxicol In Vitro. 2021; 10.1016/j.tiv.2021.105178 10.1016/j.tiv.2021.105178 33905840

[36] Kuehn A, et al. Human alveolar epithelial cells expressing tight junctions to model the air-blood barrier. Altex. 2016;33(3):251–60. 26985677 10.14573/altex.1511131

[37] Kletting S, et al. Co-culture of human alveolar epithelial (hAELVi) and macrophage (THP-1) cell lines. Altex. 2018;35(2):211–22. 29169185 10.14573/altex.1607191

[38] Carius P, et al. A monoclonal human alveolar epithelial cell line (“Arlo”) with pronounced barrier function for studying drug permeability and viral infections. Adv Sci (Weinh). 2023;10(8): e2207301. 36748276 10.1002/advs.202207301 PMC10015904

[39] Elbert KJ, et al. Monolayers of human alveolar epithelial cells in primary culture for pulmonary absorption and transport studies. Pharm Res. 1999;16(5):601–8. 10349999 10.1023/a:1018887501927

[40] Endter S, et al. RT-PCR analysis of ABC, SLC and SLCO drug transporters in human lung epithelial cell models. J Pharm Pharmacol. 2009;61(5):583–91. 19405996 10.1211/jpp/61.05.0006

[41] Bleasby K, et al. Expression profiles of 50 xenobiotic transporter genes in humans and pre-clinical species: a resource for investigations into drug disposition. Xenobiotica. 2006;36(10–11):963–88. 17118916 10.1080/00498250600861751

[42] Rotoli BM, et al. Characterization of ABC transporters in EpiAirway, a cellular model of normal human bronchial epithelium. Int J Mol Sci. 2020; 10.3390/ijms21093190 10.3390/ijms21093190 PMC7247561 32366035

[43] Sakamoto A, et al. Quantitative expression of human drug transporter proteins in lung tissues: analysis of regional, gender, and interindividual differences by liquid chromatography-tandem mass spectrometry. J Pharm Sci. 2013;102(9):3395–406. 23670800 10.1002/jps.23606

[44] Hubatsch I, Ragnarsson EG, Artursson P. Determination of drug permeability and prediction of drug absorption in Caco-2 monolayers. Nat Protoc. 2007;2(9):2111–9. 17853866 10.1038/nprot.2007.303

[45] Poirier A, et al. Calibration of in vitro multidrug resistance protein 1 substrate and inhibition assays as a basis to support the prediction of clinically relevant interactions in vivo. Drug Metab Dispos. 2014;42(9):1411–22. 24939652 10.1124/dmd.114.057943

[46] Selo MA, et al. Tobacco smoke and inhaled drugs alter expression and activity of multidrug resistance-associated protein-1 (MRP1) in human distal lung epithelial cells in vitro. Front Bioeng Biotechnol. 2020;8:1030. 33015009 10.3389/fbioe.2020.01030 PMC7505930

[47] Molenda N, et al. Paracellular transport through healthy and cystic fibrosis bronchial epithelial cell lines–do we have a proper model? PLoS ONE. 2014;9(6): e100621. 24945658 10.1371/journal.pone.0100621 PMC4063962

[48] Sakamoto A, et al. Drug transporter protein quantification of immortalized human lung cell lines derived from tracheobronchial epithelial cells (Calu-3 and BEAS2-B), bronchiolar-alveolar cells (NCI-H292 and NCI-H441), and alveolar type II-like cells (A549) by liquid chromatography-tandem mass spectrometry. J Pharm Sci. 2015;104(9):3029–38. 25690838 10.1002/jps.24381

[49] Hutter V, et al. Digoxin net secretory transport in bronchial epithelial cell layers is not exclusively mediated by P-glycoprotein/MDR1. Eur J Pharm Biopharm. 2014;86(1):74–82. 23816640 10.1016/j.ejpb.2013.06.010

[50] Hernandez-Lozano I, et al. Influence of ABC transporters on the excretion of ciprofloxacin assessed with PET imaging in mice. Eur J Pharm Sci. 2021;163: 105854. 33865975 10.1016/j.ejps.2021.105854

[51] Al-Jayyoussi G, et al. Selectivity in the impact of P-glycoprotein upon pulmonary absorption of airway-dosed substrates: a study in ex vivo lung models using chemical inhibition and genetic knockout. J Pharm Sci. 2013;102(9):3382–94. 23670704 10.1002/jps.23587

[52] Ehrhardt C, et al. 16HBE14o- human bronchial epithelial cell layers express P-glycoprotein, lung resistance-related protein, and caveolin-1. Pharm Res. 2003;20(4):545–51. 12739760 10.1023/a:1023230328687

[53] Jouan E, et al. The mitochondrial fluorescent dye rhodamine 123 is a high-affinity substrate for organic cation transporters (OCTs) 1 and 2. Fundam Clin Pharmacol. 2014;28(1):65–77. 22913740 10.1111/j.1472-8206.2012.01071.x

[54] Salomon JJ, et al. Transport of the fluorescent organic cation 4-(4-(dimethylamino)styryl)-N-methylpyridinium iodide (ASP+) in human respiratory epithelial cells. Eur J Pharm Biopharm. 2012;81(2):351–9. 22426135 10.1016/j.ejpb.2012.03.001

[55] Cho SK, et al. Verapamil decreases the glucose-lowering effect of metformin in healthy volunteers. Br J Clin Pharmacol. 2014;78(6):1426–32. 25060604 10.1111/bcp.12476 PMC4256631

[56] Hamilton KO, et al. P-glycoprotein efflux pump expression and activity in Calu-3 cells. J Pharm Sci. 2001;90(5):647–58. 11288109 10.1002/1520-6017(200105)90:5<647::aid-jps1021>3.0.co;2-g

[57] Qadir M, et al. Cyclosporin A is a broad-spectrum multidrug resistance modulator. Clin Cancer Res. 2005;11(6):2320–6. 15788683 10.1158/1078-0432.CCR-04-1725

[58] Zhou S, Zeng S, Shu Y. Drug-drug interactions at organic cation transporter 1. Front Pharmacol. 2021;12: 628705. 33679412 10.3389/fphar.2021.628705 PMC7925875

[59] Izumi S, et al. Experimental and modeling evidence supporting the trans-inhibition mechanism for preincubation time-dependent, long-lasting inhibition of organic anion transporting polypeptide 1B1 by cyclosporine A. Drug Metab Dispos. 2022;50(5):541–51. 35241487 10.1124/dmd.121.000783

[60] Salomon JJ, Ehrhardt C. Nanoparticles attenuate P-glycoprotein/MDR1 function in A549 human alveolar epithelial cells. Eur J Pharm Biopharm. 2011;77(3):392–7. 21093586 10.1016/j.ejpb.2010.11.009

[61] Visigalli R, et al. Expression and function of ABC transporters in human alveolar epithelial cells*.* Biomolecules. 2022; 10.3390/biom12091260 10.3390/biom12091260 PMC9496151 36139099

[62] Barosova H, et al. Use of EpiAlveolar lung model to predict fibrotic potential of multiwalled carbon nanotubes. ACS Nano. 2020;14(4):3941–56. 32167743 10.1021/acsnano.9b06860

[63] Endter S, et al. P-glycoprotein (MDR1) functional activity in human alveolar epithelial cell monolayers. Cell Tissue Res. 2007;328(1):77–84. 17165089 10.1007/s00441-006-0346-6

[64] US Department of Health and Human Services, Food and Drug Administration, Center for Drug Evaluation and Research (CDER), Center for Biologics Evaluation and Research (CBER). M12 Drug Interaction Studies Guidance for Industry. In: US FDA Website [online] 2024. https://www.fda.gov/media/161199/download. Accessed 23 Dec 2024.

[65] Pedersen JM, et al. Prediction and identification of drug interactions with the human ATP-binding cassette transporter multidrug-resistance associated protein 2 (MRP2; ABCC2). J Med Chem. 2008;51(11):3275–87. 18457386 10.1021/jm7015683

[66] Cole SP. Multidrug resistance protein 1 (MRP1, ABCC1), a “multitasking” ATP-binding cassette (ABC) transporter. J Biol Chem. 2014;289(45):30880–8. 25281745 10.1074/jbc.R114.609248 PMC4223294

[67] van der Deen M, et al. Cigarette smoke extract affects functional activity of MRP1 in bronchial epithelial cells. J Biochem Mol Toxicol. 2007;21(5):243–51. 17912704 10.1002/jbt.20187

[68] Hamilton KO, et al. Multidrug resistance-associated protein-1 functional activity in Calu-3 cells. J Pharmacol Exp Ther. 2001;298(3):1199–205. 11504821

[69] Imai Y, et al. Breast cancer resistance protein exports sulfated estrogens but not free estrogens. Mol Pharmacol. 2003;64(3):610–8. 12920197 10.1124/mol.64.3.610

[70] Izumi S, et al. Substrate-dependent inhibition of organic anion transporting polypeptide 1B1: comparative analysis with prototypical probe substrates estradiol-17beta-glucuronide, estrone-3-sulfate, and sulfobromophthalein. Drug Metab Dispos. 2013;41(10):1859–66. 23920221 10.1124/dmd.113.052290

[71] Karlgren M, et al. Classification of inhibitors of hepatic organic anion transporting polypeptides (OATPs): influence of protein expression on drug-drug interactions. J Med Chem. 2012;55(10):4740–63. 22541068 10.1021/jm300212s PMC3361267

[72] Sake JA, et al. Knockout of ABCC1 in NCI-H441 cells reveals CF to be a suboptimal substrate to study MRP1 activity in organotypic in vitro models. Eur J Pharm Sci. 2022;181: 106364. 36563915 10.1016/j.ejps.2022.106364

[73] Mairinger S, et al. Assessing the activity of multidrug resistance-associated protein 1 at the lung epithelial barrier. J Nucl Med. 2020;61(11):1650–7. 32284394 10.2967/jnumed.120.244038

[74] Legrand O, et al. Pgp and MRP activities using calcein-AM are prognostic factors in adult acute myeloid leukemia patients. Blood. 1998;91(12):4480–8. 9616142

[75] Torky ARW. Expression und Funktion von MRP-Proteinen als Xenobiotika-Detoxifikationssystem in humanen Lungenzellen in Kultur. Martin-Luther-University Halle-Wittenberg: Universitäts- und Landesbibliothek Sachsen-Anhalt. 2005: 10.25673/2460

[76] Poirier A, et al. The need for human breast cancer resistance protein substrate and inhibition evaluation in drug discovery and development: why, when, and how? Drug Metab Dispos. 2014;42(9):1466–77. 24989889 10.1124/dmd.114.058248

[77] Paturi DK, et al. Identification and functional characterization of breast cancer resistance protein in human bronchial epithelial cells (Calu-3). Int J Pharm. 2010;384(1–2):32–8. 19782742 10.1016/j.ijpharm.2009.09.037 PMC2830792

[78] Tang F, et al. Bidirectional transport of rhodamine 123 and Hoechst 33342, fluorescence probes of the binding sites on P-glycoprotein, across MDCK-MDR1 cell monolayers. J Pharm Sci. 2004;93(5):1185–94. 15067695 10.1002/jps.20046

[79] Chen H, et al. Elacridar, a third-generation ABCB1 inhibitor, overcomes resistance to docetaxel in non-small cell lung cancer. Oncol Lett. 2017;14(4):4349–54. 28959367 10.3892/ol.2017.6678 PMC5607652

[80] Nickel S, et al. Expression and activity of breast cancer resistance protein (BCRP/ABCG2) in human distal lung epithelial cells in vitro. Pharm Res. 2017;34(12):2477–87. 28470471 10.1007/s11095-017-2172-9

[81] Galetti M, et al. Effect of ABCG2/BCRP Expression on efflux and uptake of gefitinib in NSCLC cell lines. PLoS ONE. 2015;10(11): e0141795. 26536031 10.1371/journal.pone.0141795 PMC4633241

[82] Grainger CI, et al. Culture of Calu-3 cells at the air interface provides a representative model of the airway epithelial barrier. Pharm Res. 2006;23(7):1482–90. 16779708 10.1007/s11095-006-0255-0

[83] Stentebjerg-Andersen A, et al. Calu-3 cells grown under AIC and LCC conditions: implications for dipeptide uptake and transepithelial transport of substances. Eur J Pharm Biopharm. 2011;78(1):19–26. 21195173 10.1016/j.ejpb.2010.12.030

[84] Hayeshi R, et al. Comparison of drug transporter gene expression and functionality in Caco-2 cells from 10 different laboratories. Eur J Pharm Sci. 2008;35(5):383–96. 18782614 10.1016/j.ejps.2008.08.004

[85] Gonzalez-Mariscal L, Nava P, Hernandez S. Critical role of tight junctions in drug delivery across epithelial and endothelial cell layers. J Membr Biol. 2005;207(2):55–68. 16477528 10.1007/s00232-005-0807-y

[86] Forbes B. Human airway epithelial cell lines for in vitro drug transport and metabolism studies. Pharm Sci Technol Today. 2000;3(1):18–27. 10637597 10.1016/s1461-5347(99)00231-x

[87] Ozeki K, et al. Evaluation of the appropriate time range for estimating the apparent permeability coefficient (P(app)) in a transcellular transport study. Int J Pharm. 2015;495(2):963–71. 26387619 10.1016/j.ijpharm.2015.09.035

[88] Saaby L, Brodin B. A critical view on in vitro analysis of P-glycoprotein (P-gp) transport kinetics. J Pharm Sci. 2017;106(9):2257–64. 28438535 10.1016/j.xphs.2017.04.022

[89] Brillault J, et al. P-glycoprotein-mediated transport of moxifloxacin in a Calu-3 lung epithelial cell model. Antimicrob Agents Chemother. 2009;53(4):1457–62. 19188390 10.1128/AAC.01253-08 PMC2663113

[90] Bednarczyk D, Sanghvi MV. The impact of assay recovery on the apparent permeability, a function of lysosomal trapping. Xenobiotica. 2020;50(7):753–60. 31701802 10.1080/00498254.2019.1691284

[91] Forbes B, Lansley AB. Differences in drug transport across bronchial and gastrointestinal drug absorption models. J Pharm Pharmacol. 1998;50:96.

[92] Fischer H, Widdicombe JH. Mechanisms of acid and base secretion by the airway epithelium. J Membr Biol. 2006;211(3):139–50. 17091214 10.1007/s00232-006-0861-0 PMC2929530

[93] Ehrhardt C, et al. Current progress toward a better understanding of drug disposition within the lungs: summary proceedings of the first workshop on drug transporters in the lungs. J Pharm Sci. 2017;106(9):2234–44. 28416418 10.1016/j.xphs.2017.04.011

[94] Twentyman PR, Rhodes T, Rayner S. A comparison of rhodamine 123 accumulation and efflux in cells with P-glycoprotein-mediated and MRP-associated multidrug resistance phenotypes. Eur J Cancer. 1994;30A(9):1360–9. 7999426 10.1016/0959-8049(94)90187-2

[95] Jouan E, et al. Evaluation of P-glycoprotein inhibitory potential using a rhodamine 123 accumulation assay. Pharmaceutics, 2016; 10.3390/pharmaceutics8020012 10.3390/pharmaceutics8020012 PMC4932475 27077878

[96] Ong HX, et al. Ciprofloxacin is actively transported across bronchial lung epithelial cells using a Calu-3 air interface cell model. Antimicrob Agents Chemother. 2013;57(6):2535–40. 23507281 10.1128/AAC.00306-13 PMC3716180

[97] Ohashi R, et al. Molecular and physiological evidence for multifunctionality of carnitine/organic cation transporter OCTN2. Mol Pharmacol. 2001;59(2):358–66. 11160873 10.1124/mol.59.2.358

[98] Mease K, et al. Differential selectivity of efflux transporter inhibitors in Caco-2 and MDCK-MDR1 monolayers: a strategy to assess the interaction of a new chemical entity with P-gp, BCRP, and MRP2. J Pharm Sci. 2012;101(5):1888–97. 22359351 10.1002/jps.23069

[99] Shen F, et al. Dynamic assessment of mitoxantrone resistance and modulation of multidrug resistance by valspodar (PSC833) in multidrug resistance human cancer cells. J Pharmacol Exp Ther. 2009;330(2):423–9. 19423841 10.1124/jpet.109.153551 PMC2713081

[100] Brillault J, De Castro WV, Couet W. Relative contributions of active mediated transport and passive diffusion of fluoroquinolones with various lipophilicities in a Calu-3 lung epithelial cell model. Antimicrob Agents Chemother. 2010;54(1):543–5. 19822706 10.1128/AAC.00733-09 PMC2798514

[101] Tivnan A, et al. Inhibition of multidrug resistance protein 1 (MRP1) improves chemotherapy drug response in primary and recurrent glioblastoma multiforme. Front Neurosci. 2015;9:218. 26136652 10.3389/fnins.2015.00218 PMC4468867

[102] Myint K, et al. Multidrug resistance-associated protein 2 (MRP2) mediated transport of oxaliplatin-derived platinum in membrane vesicles. PLoS ONE. 2015;10(7): e0130727. 26131551 10.1371/journal.pone.0130727 PMC4488857

[103] Letschert K, et al. Vectorial transport of the peptide CCK-8 by double-transfected MDCKII cells stably expressing the organic anion transporter OATP1B3 (OATP8) and the export pump ABCC2. J Pharmacol Exp Ther. 2005;313(2):549–56. 15665139 10.1124/jpet.104.081224

[104] Yin J, Zhang J. Multidrug resistance-associated protein 1 (MRP1/ABCC1) polymorphism: from discovery to clinical application. Zhong Nan Da Xue Xue Bao Yi Xue Ban. 2011;36(10):927–38. 22086004 10.3969/j.issn.1672-7347.2011.10.002 PMC4297474

[105] Weidner LD, et al. The inhibitor Ko143 is not specific for ABCG2. J Pharmacol Exp Ther. 2015;354(3):384–93. 26148857 10.1124/jpet.115.225482 PMC4538874

[106] Wang Q, et al. Application and limitation of inhibitors in drug-transporter interactions studies. Int J Pharm. 2008;356(1–2):12–8. 18272304 10.1016/j.ijpharm.2007.12.024

[107] Hittinger M, et al. Preclinical safety and efficacy models for pulmonary drug delivery of antimicrobials with focus on in vitro models. Adv Drug Deliv Rev. 2015;85:44–56. 25453270 10.1016/j.addr.2014.10.011

[108] Miller AJ, Spence JR. In vitro models to study human lung development, disease and homeostasis. Physiology (Bethesda). 2017;32(3):246–60. 28404740 10.1152/physiol.00041.2016 PMC6148341

[109] Primavessy D, et al. Pulmonary in vitro instruments for the replacement of animal experiments. Eur J Pharm Biopharm. 2021;168:62–75. 34438019 10.1016/j.ejpb.2021.08.005

[110] Bas A, et al. Understanding the development, standardization, and validation process of alternative in vitro test methods for regulatory approval from a researcher perspective. Small. 2021;17(15): e2006027. 33480475 10.1002/smll.202006027

[111] Crapo JD, et al. Cell number and cell characteristics of the normal human lung. Am Rev Respir Dis. 1982;126(2):332–7. 7103258 10.1164/arrd.1982.126.2.332

[112] Abbott NJ, et al. Structure and function of the blood-brain barrier. Neurobiol Dis. 2010;37(1):13–25. 19664713 10.1016/j.nbd.2009.07.030

[113] Gehr P, Bachofen M, Weibel ER. The normal human lung: ultrastructure and morphometric estimation of diffusion capacity. Respir Physiol. 1978;32(2):121–40. 644146 10.1016/0034-5687(78)90104-4

[114] Cordon-Cardo C, et al. Expression of the multidrug resistance gene product (P-glycoprotein) in human normal and tumor tissues. J Histochem Cytochem. 1990;38(9):1277–87. 1974900 10.1177/38.9.1974900

[115] Berg T, et al. Expression of MATE1, P-gp, OCTN1 and OCTN2, in epithelial and immune cells in the lung of COPD and healthy individuals. Respir Res. 2018;19(1):68. 29678179 10.1186/s12931-018-0760-9 PMC5910606

[116] Lagowala DA, et al. Human microphysiological models of airway and alveolar epithelia. Am J Physiol Lung Cell Mol Physiol. 2021;321(6):L1072–88. 34612064 10.1152/ajplung.00103.2021 PMC8715018

[117] Stucki AO, et al. A lung-on-a-chip array with an integrated bio-inspired respiration mechanism. Lab Chip. 2015;15(5):1302–10. 25521475 10.1039/c4lc01252f

[118] Stucki JD, et al. Medium throughput breathing human primary cell alveolus-on-chip model. Sci Rep. 2018;8(1):14359. 30254327 10.1038/s41598-018-32523-x PMC6156575

